# Diagnostic chest X-rays and breast cancer risk among women with a hereditary predisposition to breast cancer unexplained by a *BRCA1* or *BRCA2* mutation

**DOI:** 10.1186/s13058-021-01456-1

**Published:** 2021-08-03

**Authors:** Maximiliano Ribeiro Guerra, Juliette Coignard, Séverine Eon-Marchais, Marie-Gabrielle Dondon, Dorothée Le Gal, Juana Beauvallet, Noura Mebirouk, Muriel Belotti, Olivier Caron, Marion Gauthier-Villars, Isabelle Coupier, Bruno Buecher, Alain Lortholary, Jean-Pierre Fricker, Paul Gesta, Catherine Noguès, Laurence Faivre, Pascaline Berthet, Elisabeth Luporsi, Capucine Delnatte, Valérie Bonadona, Christine M. Maugard, Pascal Pujol, Christine Lasset, Michel Longy, Yves-Jean Bignon, Claude Adenis-Lavignasse, Laurence Venat-Bouvet, Hélène Dreyfus, Laurence Gladieff, Isabelle Mortemousque, Séverine Audebert-Bellanger, Florent Soubrier, Sophie Giraud, Sophie Lejeune-Dumoulin, Jean-Marc Limacher, Jean Chiesa, Anne Fajac, Anne Floquet, François Eisinger, Julie Tinat, Sandra Fert-Ferrer, Chrystelle Colas, Thierry Frebourg, Francesca Damiola, Laure Barjhoux, Eve Cavaciuti, Sylvie Mazoyer, Anne Tardivon, Fabienne Lesueur, Dominique Stoppa-Lyonnet, Nadine Andrieu

**Affiliations:** 1 INSERM, U900, Paris, France; 2 Institut Curie, Paris, France; 3 Mines ParisTech, Fontainebleau, France; 4 PSL Research University, Paris, France; 5 Department of Public Health, Faculty of Medicine, Federal University of Juiz de Fora - UFJF, Minas Gerais, Brazil; 6 Institut Curie, Service de Génétique, Paris, France; 7 Gustave Roussy, Département de Médecine Oncologique, Université Paris-Saclay, Villejuif, France; 8 Hôpital Arnaud de Villeneuve, CHU Montpellier, Service de Génétique Médicale et Oncogénétique, Montpellier, France; 9 INSERM 896, CRCM Val d’Aurelle, Montpellier, France; 10 Centre Catherine de Sienne, Service d’Oncologie Médicale, Nantes, France; 11 Centre Paul Strauss, Unité d’Oncologie, Strasbourg, France; 12 CH Georges Renon, Service d’Oncogénétique Régional Poitou-Charentes, Niort, France; 13 Département d’Anticipation et de Suivi des Cancers, Oncogénétique Clinique, Institut Paoli Calmettes, Marseille, France; 14 Aix Marseille Univ, INSERM, IRD, SESSTIM, Marseille, France; 15 Institut GIMI, CHU de Dijon, Hôpital d’Enfants, Dijon, France; 16 Centre de Lutte contre le Cancer Georges François Leclerc, Dijon, France; 17 Centre François Baclesse, Unité de pathologie gynécologique, Caen, France; 18 Service de Génétique UF4128 CHR Metz-Thionville, Hôpital de Mercy, Metz, France; 19 Centre René Gauducheau, Unité d’Oncogénétique, Nantes, Saint Herblain France; 20 Université Claude Bernard Lyon 1, Villeurbanne, France; 21 CNRS UMR 5558, Lyon, France; 22 Centre Léon Bérard, Unité de Prévention et Epidémiologie Génétique, Lyon, France; 23 Génétique Oncologique moléculaire, UF1422, Département d’Oncobiologie, LBBM, Hôpitaux Universitaires de Strasbourg, Strasbourg, France; 24 UF6948 Génétique Oncologique Clinique, Evaluation familiale et suivi, Hôpitaux Universitaires de Strasbourg, Strasbourg, France; 25 Institut Bergonié, Bordeaux, France; 26 Département d’oncogénétique, Centre Jean Perrin, Université Clermont Auvergne, UMR INSERM 1240, Clermont Ferrand, France; 27 Polyclinique de la Louvière (groupe Ramsay), Lille, France; 28 Service d’Oncologie Médicale, Hôpital Universitaire Dupuytren, Limoges, France; 29 Clinique Sainte Catherine, Avignon, France; 30 Hôpital Couple-Enfant, Département de Génétique, CHU de Grenoble, Grenoble, France; 31 Institut Claudius Regaud – IUCT-Oncopole, Service d’Oncologie Médicale, Toulouse, France; 32 Service de Génétique, Hôpital Bretonneau, Tours, France; 33 Département de Génétique Médicale et Biologie de la Reproduction, Hôpital Morvan, CHU Brest, Brest, France; 34 Hôpital Tenon, Paris, France; 35 Hospices Civils de Lyon, Service de Génétique, Groupement Hospitalier EST, Bron, France; 36 Clinique de Génétique Médicale Guy Fontaine, CHU Lille, Lille, France; 37 Service d’Onco-hématologie, Hôpital Pasteur, Colmar, France; 38 Service d’Oncologie Médicale, CHRU Hôpital Caremeau, Nîmes, France; 39 Service d’Oncogénétique, Hôpital Tenon, Paris, France; 40 Groupe Hospitalier Pellegrin, Service de génétique médicale, CHU De Bordeaux, Bordeaux, France; 41 Centre Hospitalier Métropole Savoie, Chambéry, France; 42 Institut Curie, Hopital René Huguenin, Saint-Cloud, France; 43 Département de Génétique, Hopital Universitaire de Rouen, Rouen, France; 44 Department of Biopathology, Pathology Research platform, Centre Léon Bérard, Lyon, France; 45 GCS AURAGEN, Plateforme de Génétique, Hôpital Edouart Herriot, Lyon, France; 46 Centre de Recherche en Neurosciences de Lyon, INSERM U1028, CNRS UMR5292, Université Lyon 1, Université Saint Etienne, Lyon, France; 47 Service de Radiologie, Institut Curie, Paris, France; 48 INSERM, U830, Paris, France; 49 Université Paris-Descartes, Paris, France

**Keywords:** Breast cancer, X-ray exposure, Low dose, High-risk population, DNA repair genes

## Abstract

**Background:**

Diagnostic ionizing radiation is a risk factor for breast cancer (BC). BC risk increases with increased dose to the chest and decreases with increased age at exposure, with possible effect modification related to familial or genetic predisposition. While chest X-rays increase the BC risk of *BRCA1/2* mutation carriers compared to non-carriers, little is known for women with a hereditary predisposition to BC but who tested negative for a *BRCA1 or BRCA2 (BRCA1/2)* mutation.

**Methods:**

We evaluated the effect of chest X-rays from diagnostic medical procedures in a dataset composed of 1552 BC cases identified through French family cancer clinics and 1363 unrelated controls. Participants reported their history of X-ray exposures in a detailed questionnaire and were tested for 113 DNA repair genes. Logistic regression and multinomial logistic regression models were used to assess the association with BC.

**Results:**

Chest X-ray exposure doubled BC risk. A 3% increased BC risk per additional exposure was observed. Being 20 years old or younger at first exposure or being exposed before first full-term pregnancy did not seem to modify this risk. Birth after 1960 or carrying a rare likely deleterious coding variant in a DNA repair gene other than *BRCA1/2* modified the effect of chest X-ray exposure.

**Conclusion:**

Ever/never chest X-ray exposure increases BC risk 2-fold regardless of age at first exposure and, by up to 5-fold when carrying 3 or more rare variants in a DNA repair gene.

Further studies are needed to evaluate other DNA repair genes or variants to identify those which could modify radiation sensitivity. Identification of subpopulations that are more or less susceptible to ionizing radiation is important and potentially clinically relevant.

**Supplementary Information:**

The online version contains supplementary material available at 10.1186/s13058-021-01456-1.

## Introduction

Medical diagnostic ionizing radiation is a known risk factor for the development of primary breast cancer (BC). BC risk associated with exposure to such radiation increases with radiation dose and decreases with age of exposure [1, 2]. Periods of high breast cell proliferation, such as during puberty and pregnancy, are associated with increased levels of DNA synthesis and thus may make breast tissue particularly susceptible to the carcinogenic effects of radiation [1, 2]. This susceptibility to radiation may be exacerbated for women with a familial/genetic predisposition [3–7] and particularly for women carrying genetic variants altering DNA repair mechanisms that may lead to cellular radio-sensitivity [8]. Among studies conducted in the general population, few have evaluated the effect of medical radiation exposures according to family history of BC [6, 7, 9–12], and only two studies found a stronger dose response for patients with relatives affected than for patients with no family history [7, 9]. Among studies involving women carrying a *BRCA1* or *BRCA2* (*BRCA1/2*) mutation, some found an association between chest X-ray exposure and BC; almost all of these studies showed that early exposure may be a risk factor for BC [3–5, 13–18]. For women with a non-*BRCA1/2* hereditary predisposition to BC, little is known about the effect of chest radiation exposures and knowledge of such an effect may have clinical relevance. Therefore, we evaluated the effect of low-dose radiation exposure from diagnostic medical procedures on BC risk in women attending family cancer clinics, but not carrying a *BRCA1/2* mutation [19]. We also evaluated whether carrying a rare variant in a DNA repair gene other than *BRCA1/2* modified the effect of chest X-ray exposure.

## Methods

### Study population

**Fig. 1:**
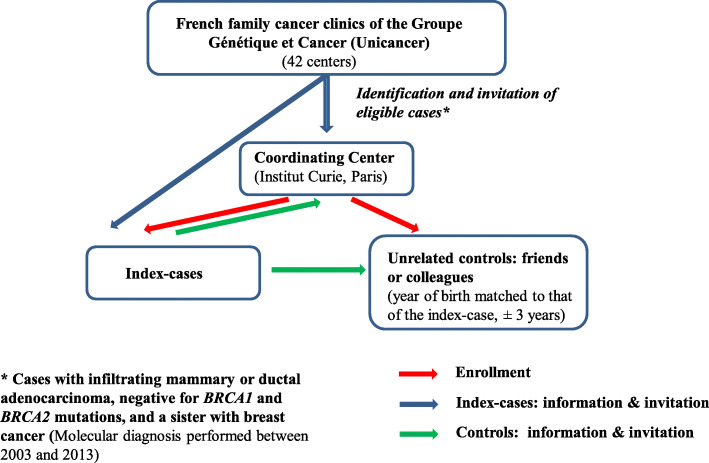
Recruitment process for index cases and unrelated controls

The GENESIS (for GENE SISter) study was initially set up to investigate the missing heritability of BC in a high-risk population with unrelated controls for conducting association studies [19]. GENESIS involved the recruitment of a study population enriched for susceptibility factors by case selection based on familial criteria (Supplementary Method Section), with consideration of environmental factors. Index cases were identified by the national network of family cancer clinics (Genetics and Cancer Group of UNICANCER) (i.e., 42 centers) when eligible, i.e., when diagnosed with infiltrating mammary or ductal adenocarcinoma, negative for *BRCA1* and *BRCA2* mutations, and had a sister with BC. The mutation screening strategy used was similar for all the clinics. Each family cancer clinic of the national network invited index cases to participate in the GENESIS study by letter or during consultations informing patients of their *BRCA1/2* negative results and referred them to the coordinating center (Curie Institute, Paris, France) if index cases consented to participate. Index cases contacted unrelated unaffected friends or colleagues with years of birth matched to ±3 years and invited them to participate and referred those who accepted to participate to the coordinating center. The coordinating center organized the enrollment of index cases and their unrelated controls, collection of questionnaires, family, and clinical data of participants (Fig. 1).

All women completed a questionnaire on environmental, lifestyle and reproductive factors, and family history of cancer. Blood samples were collected at participation (see Supplementary Methods Section in Additional file 1.doc). We considered only women reporting European ancestry (i.e., over 95% of the study population) for this evaluation.

### Exposure to low-dose radiation to the chest

Participants reported their history of chest X-ray exposure from diagnostic/screening medical procedures in a detailed questionnaire at the time of their recruitment. We considered procedures where the thoracic region was exposed such as conventional radiography, fluoroscopy, computed tomography, and scintigraphy (excluding mammograms). Age at exposure, number of exposures, type of procedure, and reason(s) for performing the examination were also documented.

To estimate lifetime exposure, pulmonary radiological examinations, preoperative radiological examinations, and radiological examinations of the heart and thoracic vessels were taken into account for all the reported procedures.

To exclude procedures that could have been performed because of BC diagnosis, we included exposures that occurred up to 1 year prior to BC diagnosis for cases and 1 year prior to the date of questionnaire completion for controls.

For each type of procedure, information on lifetime exposure (ever/never), number of exposures, and age at first exposure were collected. Variables considered in the analyses were ever versus never exposed, number of exposures, age at first exposure, and timing of first exposure relative to the first full-term pregnancy (FFTP).

We excluded 52 women who underwent radiotherapy for a benign disease 1 year prior to age at censoring (2.19% cases, 1.23% controls). Among cases, we also excluded 10 women (0.63%) who underwent radiotherapy for a cancer other than BC before their BC diagnosis.

### DNA repair-related variants

We previously assessed the contribution of rare germline deleterious or likely deleterious variants (with minor allele frequency >0.5% in controls) in 113 DNA repair genes in familial BC by performing targeted sequencing of the entire coding sequence in 1207 cases and 1199 controls from GENESIS. Detailed information on the selection of genes, sequencing procedure and variants filtering and annotation is described in Girard et al. [20] (see Supplementary Methods Section in Additional file 1.doc). Published results of the association tests per gene are shown in Table 1. Sequencing data were available for 82.5% of the GENESIS subjects investigated in the present study. There was no difference in the distribution of the characteristics between the subsets of cases and controls with and without sequencing data (see Supplemental Table 1 in Additional file 2.doc).

**Table 1:** Association of rare coding variants with breast cancer, for the 113 DNA repair genes sequenced in the GENESIS population [20]

Gene	Any variant				
	Control carriers	Case carriers	OR^**a**^ (95% CI)	***P*** value	Group^**b**^
***BABAM1/MERIT40***	4	2	0.5 (0.1, 2.8)	0.43	Low
***BACH1***	22	21	0.8 (0.4, 1.5)	0.55	Low
***BRCC3/BRCC36***	3	1	0.3 (0.0, 3.2)	0.34	Low
***BRE***	11	7	0.6 (0.2, 1.6)	0.35	Low
***CDH1***	14	11	0.8 (0.4, 1.7)	0.54	Low
***CDKN1A***	13	11	0.8 (0.4, 1.9)	0.66	Low
***COBRA1***	6	4	0.7 (0.2, 2.4)	0.55	Low
***DLG1***	25	15	0.6 (0.3, 1.2)	0.12	Low
***ESR1***	10	6	0.6 (0.2, 1.5)	0.26	Low
***EXO1***	45	37	0.8 (0.5, 1.3)	0.41	Low
***FAM175A/ABRAXAS***	7	5	0.7 (0.2, 2.3)	0.56	Low
***FANCA***	19	15	0.8 (0.4, 1.6)	0.53	Low
***FANCD2***	20	15	0.7 (0.4, 1.4)	0.33	Low
***FANCF***	6	5	0.8 (0.3, 2.8)	0.77	Low
***FANCG***	6	4	0.7 (0.2, 2.4)	0.55	Low
***FANCI***	22	13	0.6 (0.3, 1.2)	0.13	Low
***IRS2***	13	9	0.7 (0.3, 1.5)	0.33	Low
***KIAA1967***	22	17	0.8 (0.4, 1.5)	0.41	Low
***LIG4***	15	13	0.7 (0.3, 1.6)	0.46	Low
***MLH3***	21	10	0.5 (0.2, 1.0)	0.06	Low
***MUS81***	10	7	0.6 (0.2, 1.6)	0.30	Low
***MYC***	2	2	0.8 (0.1, 5.5)	0.78	Low
***NAT1***	3	1	0.4 (0.0, 3.4)	0.37	Low
***PMS2***	22	16	0.7 (0.3, 1.3)	0.24	Low
***POLH***	13	5	0.4 (0.1, 1.1)	0.07	Low
***POLQ***	43	36	0.8 (0.5, 1.2)	0.43	Low
***PRKAA2***	8	3	0.4 (0.1, 1.5)	0.16	Low
***RAD51D/RAD51L3***	9	4	0.4 (0.1, 1.4)	0.17	Low
***RAD54L***	21	12	0.6 (0.3, 1.2)	0.14	Low
***RTEL1***	20	8	0.4 (0.2, 0.9)	0.03	Low
***TIMELESS***	28	23	0.8 (0.5, 1.4)	0.44	Low
***TP53BP1***	27	21	0.8 (0.5, 1.5)	0.51	Low
***TP63***	6	3	0.5 (0.1, 2.0)	0.33	Low
***TTI2***	9	7	0.8 (0.3, 2.2)	0.67	Low
***WDR48***	15	10	0.7 (0.3, 1.5)	0.36	Low
***XRCC1***	31	21	0.7 (0.4, 1.2)	0.14	Low
***APEX1***	10	10	1.0 (0.4, 2.4)	0.98	No effect
***AR***	32	31	1.0 (0.6, 1.6)	0.94	No effect
***ATR***	30	29	1.0 (0.6, 1.6)	0.92	No effect
***BAP1***	4	4	1.0 (0.3, 4.0)	1.00	No effect
***BLM***	35	31	0.9 (0.5, 1.4)	0.59	No effect
***CDC27***	12	12	1.0 (0.5, 2.3)	0.98	No effect
***CDKN2A***	3	3	1.0 (0.2, 4.9)	0.99	No effect
***EIF4G1***	26	29	1.1 (0.7, 1.9)	0.67	No effect
***EP300***	21	18	0.9 (0.5, 1.6)	0.66	No effect
***ERCC6***	47	53	1.1 (0.8, 1.7)	0.55	No effect
***FANCB***	9	9	0.9 (0.4, 2.4)	0.87	No effect
***FANCC***	11	10	0.9 (0.4, 2.2)	0.87	No effect
***FANCE***	11	10	0.9 (0.4, 2.2)	0.86	No effect
***FANCL***	9	10	1.1 (0.4, 2.8)	0.84	No effect
***FLNA***	25	24	1.0 (0.6, 1.7)	0.94	No effect
***MAGI3***	26	28	1.1 (0.6, 1.8)	0.82	No effect
***MAST2***	46	50	1.1 (0.7, 1.7)	0.66	No effect
***MCM4***	25	29	1.1 (0.7, 1.9)	0.70	No effect
***MCPH1***	27	28	1.0 (0.6, 1.8)	0.92	No effect
***MDC1***	24	22	0.9 (0.5, 1.7)	0.81	No effect
***MSH2***	18	17	0.9 (0.5, 1.8)	0.76	No effect
***MSH6***	16	16	0.9 (0.5, 1.9)	0.86	No effect
***NBN***	26	27	1.0 (0.6, 1.8)	0.87	No effect
***PHLPP2***	32	34	1.0 (0.6, 1.7)	0.97	No effect
***POLK***	22	22	1.0 (0.5, 1.8)	0.99	No effect
***RAD51B/REC2/RAD51L1***	6	5	0.9 (0.3, 2.8)	0.80	No effect
***RECQL4***	49	55	1.1 (0.8, 1.7)	0.59	No effect
***RINT1***	8	8	1.0 (0.4, 2.8)	0.95	No effect
***SETX***	25	24	1.0 (0.6, 1.7)	0.91	No effect
***TELO2***	17	18	1.1 (0.5, 2.1)	0.89	No effect
***XRCC2***	7	6	0.9 (0.3, 2.7)	0.84	No effect
***APLF***	7	11	1.5 (0.6, 3.9)	0.40	High
***ATM***	40	77	1.9 (1.3, 2.9)	0.001	High
***BARD1***	7	9	1.3 (0.5, 3.6)	0.59	High
***BRIP1/FANCJ***	16	25	1.5 (0.8, 2.8)	0.25	High
***CHEK1***	4	6	1.2 (0.3, 4.5)	0.75	High
***CHEK2***	22	62	3.0 (1.9, 5.0)	0.00001	High
***CHGB***	9	11	1.2 (0.5, 3.0)	0.65	High
***DCLRE1C***	9	14	1.6 (0.7, 3.7)	0.28	High
***DGKZ***	33	38	1.2 (0.7, 1.9)	0.52	High
***ERCC2***	17	27	1.6 (0.9, 3.0)	0.13	High
***EYA3***	6	7	1.2 (0.4, 3.5)	0.77	High
***FANCM***	23	38	1.7 (1.0, 2.8)	0.06	High
***FEN1***	6	7	1.2 (0.4, 3.6)	0.74	High
***FOXO1***	6	7	1.8 (0.5, 6.0)	0.38	High
***FOXO3***	0	8	-	-	High
***FOXO4***	0	4	-	-	High
***MAST1***	8	17	2.2 (0.9, 5.1)	0.07	High
***MCM7***	10	18	1.8 (0.8, 4.0)	0.13	High
***MLH1***	15	19	1.3 (0.6, 2.5)	0.52	High
***MRE11A***	12	14	1.2 (0.6, 2.6)	0.64	High
***MSH3***	25	30	1.2 (0.7, 2.1)	0.49	High
***NTHL1***	18	22	1.2 (0.6, 2.2)	0.65	High
***NUMA1***	36	51	1.4 (0.9, 2.2)	0.12	High
***PALB2***	9	30	3.5 (1.7, 7.5)	0.001	High
***PIK3R1***	1	4	4.3 (0.5, 38.3)	0.20	High
***PMS1***	6	10	1.5 (0.6, 4.3)	0.41	High
***PPM1D***	4	6	1.5 (0.4, 5.4)	0.53	High
***PTEN***	0	4	-	-	High
***RAD50***	30	37	1.2 (0.7, 2.0)	0.44	High
***RAD51C***	7	10	1.5 (0.6, 4.0)	0.41	High
***RAD9B***	4	6	1.5 (0.4, 5.2)	0.55	High
***RECQL5***	20	29	1.5 (0.9, 2.7)	0.14	High
***REV3L***	31	39	1.3 (0.8, 2.1)	0.30	High
***RNF168***	13	16	1.2 (0.6, 2.6)	0.59	High
***RPA1***	9	14	1.5 (0.7, 3.6)	0.32	High
***SLX4/FANCP***	36	44	1.2 (0.8, 1.9)	0.38	High
***STK11***	1	2	2.1 (0.2, 22.9)	0.55	High
***TGFB1***	5	9	1.6 (0.5, 4.9)	0.38	High
***TOP3A***	22	31	1.4 (0.8, 2.5)	0.23	High
***TP53***	3	6	2.0 (0.5, 8.0)	0.34	High
***TSC2***	45	56	1.3 (0.9, 1.9)	0.23	High
***TTI1***	26	30	1.2 (0.7, 2.0)	0.57	High
***UIMC1/RAP80***	12	15	1.2 (0.6, 2.7)	0.58	High
***USP8***	9	16	1.7 (0.7, 3.8)	0.23	High
***WRN***	47	59	1.3 (0.9, 1.9)	0.23	High
***XRCC3***	4	7	1.8 (0.5, 6.2)	0.36	High

Abbreviations: OR (95%CI) odds ratio (95% confidence interval)

^a^Reference group: non-carrier of a variant in the tested gene

^**b**^Group “Low”: OR <0.9; Group “No Effect”: 0.9≤ OR ≤1.1; Group “High”: OR >1.1

Because each gene has very low deleterious or likely deleterious variant frequencies (frequency of the pool of variants for each gene ranged between 0% and 4.1% in controls) and thereby stratification by X-ray exposure and by gene led to very small numbers of subjects or even no subjects, we grouped the genes according to the value of their association with BC, i.e., the odds ratio (OR) point estimate obtained in the study by Girard et al. (Table 1) and classified them as follows: Group “Low” including genes with OR<0.9; Group “No Effect,” including genes with 0.9≤OR≤1.1 and Group “High,” including genes with OR>1.1. An individual could be assigned to more than one group if carrying variants in genes belonging to different groups.

### Statistical analyses

To assess the association between chest X-ray exposure and risk of BC, we used logistic regression models. To assess whether the association varied according to tumor estrogen receptors (ER) status, we used multinomial regression models. Analyses were adjusted for age at censoring, which was calculated as the age at diagnosis for cases, and the age at interview for controls. Other adjustment variables were education level (not graduated, basic level, intermediate/high level), birth cohort (≤1945, 1946–1959, ≥1960), body mass index at diagnosis for cases, and at interview for controls (<18.5, 18.5–24.99, 25–29.99, ≥30), number of full-term pregnancies (nulliparous, 1–2, >2), age at FFTP (<20, 20–24, 25–29, ≥30), mammography exposure at least 1 year before censoring (ever/never), and family history of BC. For this latter variable, the number of first- or second-degree relatives affected with BC was generated. Since cases had an affected sister by design, we excluded one affected sister from the family history count to assess cases’ BC family history distribution unbiased by the study design and classified BC family history as none affected, at least one 1st degree relative affected, or only 2nd degree relatives affected. We also adjusted for the number of chest X-ray exposures (≤5 vs. >5) when appropriate.

We assessed associations by birth cohort, age at censor, family history of BC, and DNA repair gene group; we used likelihood ratio tests to test for heterogeneity. Additionally, we adjusted for other gene groups when the analysis was stratified by the gene group.

We assessed heterogeneity between estrogen receptor (ER) tumor status using a multinomial logistic regression model and tested equality of coefficients between equations by difference between the log-likelihoods assuming a chi-square distribution with 1 degree of freedom (df) for never/ever exposed, 2 df for timing to first full-term pregnancy (FFTP) and age at first exposure, and 3 df for number of exposures*.*

To minimize potential survival bias, we also conducted an analysis restricted to cases diagnosed at most 5 years before enrollment in GENESIS.

Finally, as the DNA repair gene groups were defined using a priori bounds for ORs, we performed sensitivity analyses using different bounds (i.e., Group “Low”: OR<0.8 or <1.0; Group “No Effect”: 0.8≤OR≤1.2 or OR=1.0; Group “High”: OR>1.2 or >1.0).

To evaluate the effect of missing information on the observed results, we performed multiple imputations using the chained equations method (MICE) [21, 22] as implemented in STATA [23]. This method uses a Gibbs-like algorithm [24] to obtain 100 imputed datasets with complete observations for each outcome. ORs estimated on the imputed data sets were pooled together using Rubin’s rules to obtain valid statistical inferences [25].

All *P* values were two-sided and a 5% level of significance was used. All analyses were performed using Stata software version 14 [23].

## Results

Characteristics of the study population are described in Table 2. Most of the cases were prevalent with a mean delay between diagnosis and interview of 8.3 years (SD:±7.1). The mean age at BC diagnosis was 50.2 years (SD:±9.3), and the mean age at interview for the controls was 55.8 years (SD:±9.9).

**Table 2:** Characteristics of GENESIS participants

Characteristics	Cases *N* = 1,552		Controls *N* = 1,363	
	No.	%	No.	%
**Birth cohort**				
≤1945	488	31.4	294	21.6
1946–59	797	51.4	706	51.8
≥1960	267	17.2	363	26.6
**Age at censoring, years**				
Mean (sd)	50.2 (9.3)		55.8 (9.9)	
≤45	513	33.1	201	14.8
46–50	336	21.7	197	14.5
51–60	473	30.5	485	35.6
>60	230	14.8	480	35.2
**Education level**				
Intermediate/high	780	50.3	916	67.2
Basic	714	46.0	434	31.8
Not graduated	58	3.7	12	0.9
Missing	0	0.0	1	0.1
**Body mass index**				
18.5–24.9	1,019	65.6	869	63.8
<18.5	69	4.5	32	2.3
≥25 and <30	341	22.0	345	25.3
≥30	120	7.7	117	8.6
Missing	3	0.2	0	0.0
**Smoking**				
No	832	53.6	680	49.9
Current	159	10.2	158	11.6
Past	550	35.4	514	37.7
Missing	11	0.7	11	0.8
**Number of full term pregnancies**				
≥2	445	28.7	411	30.2
1–2	921	59.3	763	56.0
0	185	11.9	187	13.7
Missing	1	0.1	2	0.1
**Age at first full-term pregnancy, years**				
<20	179	11.5	102	7.5
20–24	628	40.5	531	39.0
25–29	393	25.4	392	28.8
≥30	165	10.6	149	10.9
No full-term pregnancy	185	11.9	187	13.7
Missing	2	0.1	2	0.1
**Family history of breast cancer**^**a**^				
None	427	27.5	959	70.4
1st degree	818	52.7	187	13.7
Only 2nd degree	307	19.8	216	15.9
Missing	0	0.0	1	0.1
**Tumor estrogen receptors (ER)**				
ER+	818	52.7		
ER-	168	10.8		
Missing	566	36.5		
**Gene group**^**b**^				
Group “Low”				
0	723	46.6	721	52.9
> 1	274	17.7	441	32.4
Group “No Effect”				
0	575	37.1	659	48.4
>1	422	27.2	503	36.9
Group “High”				
0	422	27.2	667	48.9
>1	575	37.1	495	36.3
Missing	555	35.8	201	14.8

^a^Excluding one affected sister per index case, “none” means no history of BC for controls or no additional BC case in the family for cases; “1st degree” means 1st degree family history for controls or additional 1st degree relative for cases and “2nd degree” means only 2nd degree family history for controls or only additional 2nd degree family history for cases

^**b**^Individuals carrying at least one variant in one of the Gene Groups: Group “Low” (OR<0.9); Group “No Effect” (0.9≤OR≤1.1); Group “High” (OR>1.1)

Compared to controls, cases were more likely to have a basic education level, lower BMI, younger age at the FFTP, and as expected, stronger family history of BC. Regarding birth cohort, cases were more likely to be born before 1945 than controls. Among the subset of participants who were sequenced for the 113 DNA repair genes (74.1%), 20% of women did not carry any variant and 30.9% of women carried only one variant. Among those who carried at least two variants, 21.9% of them carried variants in the same group, and 14.7% carried variants in genes from all three groups (data not shown). Cases carried variants from Group “High” more often than controls (57.7% and 42.6%, respectively) (Table 2).

The distribution of chest X-ray exposures, by type of medical procedure, is shown in Table 3. When considering conventional radiography plus fluoroscopy, the mean age at first chest X-ray exposure was significantly lower for cases than for controls (20.4 and 22.0 years, respectively; *P*=0.003) with a higher percentage of cases exposed before age 20 years than controls (37.0% and 31.9%, respectively, *P*<10^-3^; data not shown).

**Table 3:** Chest diagnostic/screening X-ray exposure characteristics by medical procedures

Characteristics^**a**^	Cases *N* = 1552		Controls *N* = 1363	
	No.	%	No.	%
**Conventional radiography + fluoroscopy**				
Never	213	13.7	242	17.8
Ever	1,296	83.5	1,097	80.5
Missing	43	2.8	24	1.8
Number of lifetime exposures				
1–3	377	24.3	379	27.8
4–9	248	16.0	195	14.3
≥10	258	16.6	213	15.6
Missing	456	29.4	334	24.5
Age at first exposure (years)				
Mean (sd)	20.4 (10.9)		22.0 (12.5)	
Missing	291	18.8	202	14.8
**Tomography**				
Never	1,389	89.5	1,276	93.6
Ever	48	3.1	40	2.9
Missing	115	7.4	47	3.5
Number of lifetime exposures				
1	37	2.4	30	2.2
≥2	9	0.6	9	0.7
Missing	117	7.5	48	3.5
Age at first exposure (years)				
Mean (sd)	40.0 (15.8)		42.8 (15.4)	
Missing	115	7.4	47	3.5
**Scintigraphy**				
Never	1,416	91.2	1,294	94.9
Ever	21	1.4	22	1.6
Missing	115	7.4	47	3.5
Number of lifetime exposures				
1	18	1.2	18	1.3
≥2	3	0.2	4	0.3
Missing	115	7.4	47	3.5
Age at first exposure (years)				
Mean (sd)	50.8 (10.2)		53.4 (12.0)	
Missing	115	7.4	47	3.5
**Mammography**				
Never	266	17.1	102	7.5
Ever	1271	81.9	1252	91.9
Missing	15	1.0	9	0.7

^**a**^ Lifetime exposures up to one year prior to diagnosis for cases and up to one year prior to date of questionnaire completion for controls

We found that exposure to chest X-rays was associated with a 2-fold increased odds of BC (*P*<10^-3^) compared to non-exposed women (Table 4). Each additional procedure was associated with a 3% increased odds of BC (*P*<10^-3^).

**Table 4:** Effect of lifetime chest X-ray exposure (any exposure) on breast cancer risk according to the number of exposures, the age at first exposure, and the first full-term pregnancy

	Number of				Multiple Imputation	
	Cases	Controls	OR^**a**^	95% CI	OR^**a**^	95% CI
**Chest X-ray exposure**^b^						
Never	208	239	1		1	
Ever	1304	1104	2.05	1.55–2.73	2.05	1.54–2.72
**Number of exposures**						
0	208	239	1		1	
1–3	392	390	1.70	1.23–2.34	1.62	1.18–2.23
4–9	251	200	2.52	1.76–3.61	2.29	1.65–3.16
≥10	263	215	2.37	1.64–3.43	2.70	1.89–3.87
Continuous			1.03	1.01–1.04	1.03	1.02–1.05
**Age at first exposure, years**^c^						
No exposure	208	239	0.55	0.40–0.76	0.58	0.42–0.80
≥20	485	490	1		1	
15–19	288	222	1.02	0.75–1.37	1.01	0.75–1.36
<15	290	219	1.11	0.83–1.50	1.08	0.80–1.45
Continuous			1.00	0.99–1.01	1.01	0.99–1.02
**According to first full-term pregnancy (FFTP)**^c^						
Only after FFTP^d^	268	232	1		1	
Before (incl. no FTP)	825	725	0.81	0.61–1.08	0.85	0.64–1.12
**According to first FFTP and number of exposures**						
Only after and ≤5^d^	186	178	1		1	
Only after and >5^d^	56	43	1.77	0.98–3.18	1.68	0.99–2.85
Before and ≤5 (incl. no FTP)	442	412	0.95	0.67–1.36	0.89	0.63–1.25
Before and >5 (incl. no FTP)	272	215	1.12	0.77–1.61	1.33	0.96–1.85

Abbreviations: OR (95%CI) odds ratio (95% confidence interval)

^**a**^Adjusted for age at censoring, birth cohort (≤1945; 1946–1959; ≥1960), number of full-term pregnancies (>2; 1–2; 0), mammography use (never; ever), educational level (intermediate/high; basic; not graduated), BMI (18.5–24.9; <18.5; ≥25 and <30; ≥30), smoking (no; current; past), and breast cancer family history (0;1st degree; 2nd degree)

^b^Chest X-ray exposure includes pulmonary radiological examinations in the field of preventive/occupational medicine or for lung disease, preoperative radiological examinations, and radiological examinations of heart and thoracic vessels for all the reported procedures

^c^Adjusted as in ^a ^plus number of exposures (≤5;>5)

^d^After = also includes chest X-ray exposure that occurred during the same year of first full-term pregnancy

When analyses were performed according to birth cohort, the association between chest X-ray exposure and BC risk was significant for later birth cohorts, i.e., women born after 1945 (ever vs. never, 1946–1959: OR=1.65, ≥1960: OR=2.54), with a significantly higher risk for women born after 1960 (*P*_het_=0.024) (Table 5). We also found significant heterogeneity between birth cohorts for the effect of the number of exposures on BC risk (*P*_het_=0.041) with each additional procedure associated with a 6% increased odds of BC (*P*<10^-3^) for women born after 1960. When analyses were performed according to age at censoring and family history of BC (see Supplemental Tables 2-3 in Additional file 2.doc), none of the heterogeneity tests were significant. However, the effect of chest X-ray exposure was significant only for women over the age of 60 with each additional procedure being associated with a 2% increased odds (*P*=0.036).

**Table 5:** Effect of lifetime chest X-ray exposure (any exposure) on breast cancer risk according to the number of exposures, the age at first exposure, and the first full-term pregnancy by birth cohort

	Number of				Number of				Number of				***P***^**b**^
	Cases	Ctrls	OR^**a**^	95% CI	Cases	Ctrls	OR^**a**^	95% CI	Cases	Ctrls	OR^**a**^	95% CI	
	Birth cohort												
	≤ 1945				1946–1959				≥ 1960				
**Chest X-ray exposure**													
No	47	31	1		96	92	1		65	116	1		
Yes	431	259	1.57	0.72-3.43	678	605	1.65	1.06–2.56	195	240	2.54	1.63–3.98	0.024
**Number of exposures**													
0	47	31	1		96	92	1		65	116	1		
1–3	134	81	1.72	0.74–3.98	179	197	1.24	0.75–2.05	79	112	2.04	1.22–3.40	
4–9	81	46	2.04	0.82–5.07	122	111	1.70	0.98–2.94	48	43	3.77	2.03–7.01	0.041
≥10	105	64	1.44	0.60–3.45	142	130	2.26	1.31–3.90	16	21	2.77	1.19–6.47	
Continuous			1.01	0.99–1.03			1.04	1.02–1.06			1.06	1.01–1.12	0.013
**Age at first exposure, years**^c^													
No exposure	47	31	0.52	0.23–1.21	96	92	0.77	0.47–1.25	65	116	0.47	0.28–0.78	
≥20	163	119	1		237	261	1		85	110	1		
15–19	87	45	0.74	0.38–1.42	159	123	1.07	0.69–1.66	42	54	1.13	0.64–2.00	0.67
<15	122	64	0.83	0.47–1.46	140	114	1.40	0.89–2.18	28	41	0.91	0.47–1.79	
Continuous			1.01	0.99–1.02			1.00	0.98–1.01			1.02	0.99–1.05	0.49
**According to first full-term pregnancy (FFTP)**^c^													
Only after FFTP^d^	109	59	1		124	139	1		35	34	1		
Before (incl. no FTP)	268	175	0.48	0.27–0.86	429	373	0.95	0.63–1.44	128	177	0.86	0.45–1.63	0.19

Abbreviations: OR (95% CI) odds ratio (95% confidence interval

^**a**^Adjusted for age at censoring (when by birth cohort analysis) and for birth cohort (continuous) (when by age at censure analysis), number of full-term pregnancies (>2; 1–2; 0), mammography use (never; ever), educational level (intermediate/high; basic; not graduated), BMI (18.5–24.99; <18.5; ≥25 and <30; ≥30), smoking (no; current; past), and family history of breast cancer (0;1st degree; 2nd degree)

^b^*P* value for heterogeneity test

^c^Adjusted as in ^**a**^ plus number of exposures (≤5; >5)

^d^After = also includes chest X-ray exposure that occurred during year of first full-term pregnancy

Interestingly, when stratifying on the gene group, we found that for women carrying at least one variant in Group “High” (i.e., OR>1.1) (see Supplemental Table 4 in Additional file 2.doc), the effect of chest X-ray exposures on BC risk was significantly higher than for those carrying at least one variant in the other groups (*P*_het_=0.0038) (Table 6) (ever vs. never: Group “Low” _(OR<0.9)_:OR=2.02; Group “No Effect”_(0.9≤OR≤1.1)_: OR=1.62; Group “High” _(OR>1.1)_: OR=3.31). Having had ten or more exposures also doubled the BC risk for women with a variant from Group “High” _(OR>1.1)_ compared with women in the other two groups (*P*_het_=0.022).

**Table 6:** Effect of lifetime chest X-ray exposure (any exposure) on breast cancer risk according to the number of exposures, the age at first exposure, and the first full-term pregnancy by gene group

	Number of				Number of				Number of				***P***^**b**^
	Cases	Ctrls	OR^**a**^	95% CI	Cases	Ctrls	OR^**a**^	95% CI	Cases	Ctrls	OR^**a**^	95% CI	
	Gene group												
	Group “Low”^**f**^				Group “No Effect”^**f**^				Group “High”^**f**^				
**Chest X-ray exposure**													
No	34	67	1		61	81	1		65	91	1		
Yes	234	371	2.02	1.16–3.52	354	416	1.62	1.03–2.55	495	400	3.31	2.14–5.12	0.0038
**Number of exposures**													
0	34	67	1		61	81	1		65	91	1		
<10	119	183	2.01	1.12–3.59	184	215	1.53	0.95–2.46	256	219	2.98	1.89–4.70	
≥10	47	75	2.19	1.09–4.40	67	85	2.04	1.14–3.66	94	78	3.88	2.23–6.75	0.022
Continuous			1.02	0.99–1.04			1.02	1.00–1.04			1.04	1.02–1.06	0.032
**Age at first exposure, years**^d^													
No exposure	34	67	0.55	0.30–1.01	61	81	0.83	0.51–1.37	65	91	0.38	0.23–0.61	0.43
≥20	90	163	1		124	188	1		179	180	1		
<20	101	143	1.24	0.80–1.91	166	165	1.38	0.94–2.03	222	159	1.27	0.90–1.80	
Continuous			1.00	0.9–1.02			0.99	0.98–1.01			1.00	0.98–1.01	0.78
**According to first full-term pregnancy (FFTP)**^d^													
Only after FFTP^e^	52	75	1		68	91	1		95	92	1		
Before (incl. no FTP)	142	242	0.82	0.49–1.38	231	271	0.90	0.57–1.40	311	257	1.04	0.69–1.57	0.48

Abbreviations: OR  (95% CI) odds ratio (95% confidence interval)

^**a**^Adjusted for age at censoring, birth cohort (≤1945; 1946–1959; ≥1960), number of full-term pregnancies (>2; 1–2; 0), mammography use (never; ever), educational level (intermediate/high; basic; not graduated), BMI (18.5–24.99; <18.5; ≥25 and <30; ≥30), smoking (no; current; past), plus two other DNA repair rare variants groups (when analysis by group of DNA repair rare variants), with missing included in reference categories for each variable

^b^Because of the non-exclusivity of the 3 groups**,** *P* value for heterogeneity test was performed on the ranked Group variable : at least 1 variant in Group “High,” no variant in Group “High,” and at least 1 variant in Group “No Effect,” only at least 1 variant in Group “Low”

^d^Adjusted as in ^**a**^ plus number of exposures (≤5;>5)

^e^After = also includes chest X-ray exposure that occurred during the same year of first full-term pregnancy

^f^Individuals carrying at least one variant in one of the Gene Groups: Group “Low” (OR<0.9) ; Group “No Effect” (0.9<=OR<=1.1); Group “High” (OR>1.1)

When considering chest X-ray exposure (ever vs. never) and number of variants simultaneously, BC risk increased with increasing number of variants from Group “High” _(OR>1.1)_ with a significant 66% increased odds of BC for each additional variant. Inversely, BC risk decreased with increasing number of variants from Group “Low” _(OR<0.9)_ with a significant 31% decreased odds of BC for each additional variant (Table 7).

**Table 7:** Combined effect of chest X-ray exposure and genetic variants

Chest X-ray exposure & number of DNA repair rare variants	Number of				Multiple imputation	
	Cases	Ctrls	OR^a^	95%CI	OR^a^	95%CI
**Group ‘Low’**^b^						
Ever & 0 variant	604	580	1		1	
Ever & 1 variant	197	288	0.67	0.52-0.86	0.67	0.52-0.87
Ever & 2 variants	34	69	0.52	0.32-0.84	0.49	0.30-0.79
Ever & ≥3 variants	3	14	0.25	0.06-1.00	0.25	0.06-0.99
Never	135	195	0.50	0.37-0.68	0.31	0.18-0.52
Continuous^e^			0.69	0.57-0.84	0.69	0.58-0.83
Never & 0 variant	101	128	1		1	
Never & 1 variant	27	50	0.56	0.30-1.06	0.56	0.30-1.05
Never & 2 variants	7	16	0.52	0.17-1.57	0.48	0.16-1.44
Never & ≥3 variants	0	1	-		-	
Ever & 0 variant	604	580	1.67	1.19-2.34	1.66	1.18-2.34
Ever & 1 variant	197	288	1.12	0.77-1.62	1.12	0.77-1.64
Ever & 2 variants	34	69	0.86	0.50-1.51	0.82	0.46-1.43
Ever &≥3 variants	3	14	0.42	0.10-1.71	0.42	0.10-1.71
**Group ‘No Effect’**^c^						
Ever & 0 variant	484	535	1		1	
Ever & 1 variant	258	318	0.96	0.76-1.22	0.97	0.76-1.23
Ever & 2 variants	81	78	1.12	0.76-1.65	1.15	0.77-1.70
Ever & ≥3 variants	15	20	1.21	0.55-2.70	1.28	0.57-2.87
Never	135	195	0.58	0.43-0.78	0.61	0.40-0.94
Continuous^e^			1.02	0.86-1.21	1.05	0.90-1.21
Never & 0 variant	74	114	1		1	
Never & 1 variant	45	67	1.08	0.62-1.90	1.08	0.61-1.89
Never & 2 variants	14	12	1.40	0.55-3.59	1.50	0.59-3.84
Never & ≥3 variants	2	2	1.90	0.23-15.8	1.93	0.23-16.1
Ever & 0 variant	484	535	1.85	1.27-2.70	1.85	1.27-2.71
Ever & 1 variant	258	318	1.77	1.19-2.65	1.78	1.19-2.66
Ever & 2 variants	81	78	2.07	1.25-3.42	2.13	1.29-3.54
Ever & ≥3 variants	15	20	2.24	0.95-5.32	2.39	1.01-5.64
**Group ‘High’**^d^						
Ever & 0 variant	343	551	1		1	
Ever & 1 variant	327	291	2.06	1.62-2.63	2.07	1.62-2.65
Ever & 2 variants	122	93	2.34	1.66-3.31	2.28	1.60-3.23
Ever & ≥3 variants	46	16	4.61	2.34-9.08	4.65	2.35-9.22
Never	135	195	0.85	0.63-1.16	0.74	0.49-1.13
Continuous^e^			1.67	1.42-1.96	1.66	1.44-1.90
Never & 0 variant	70	104	1		1	
Never & 1 variant	46	62	0.90	0.51-1.58	0.88	0.50-1.54
Never & 2 variants	13	23	0.66	0.27-1.57	0.58	0.24-1.41
Never & ≥3 variants	6	6	2.01	0.54-7.48	2.01	0.54-7.45
Ever & 0 variant	343	551	1.12	0.75-1.65	1.10	0.75-1.63
Ever & 1 variant	327	291	2.30	1.54-3.44	2.28	1.53-3.40
Ever & 2 variants	122	93	2.61	1.63-4.19	2.51	1.56-4.03
Ever & ≥3 variants	46	16	5.15	2.43-10.9	5.10	2.41-10.8

*Abbreviations*: *OR (95%CI)* odds ratio (95% confidence interval)

^a^Adjusted for age at censoring, birth cohort (≤1945; 1946-1959; ≥1960), number of full-term pregnancies (>2; 1-2; 0), mammography use (never; ever), educational level (intermediate/high; basic; not graduated), BMI (18.5-24.99; <18.5; ≥25 and <30; ≥30), smoking (no; current; past), and two other DNA repair genes groups

^b^at least one variant in a gene from Group ‘Low’

^c^at least one variant in a gene from Group ‘No Effect’

^d^at least one variant in a gene from Group ‘High’

^e^“Never” excluded

When analyses were performed according to tumor ER status (Table 8), the heterogeneity tests were not significant when we compared ORs between ER− and ER+ tumors for any chest X-ray exposure variables, although there was a suggestive stronger association in the ever vs. never analysis for women with ER+ tumors when compared to women with ER− tumors.

**Table 8:** Effect of lifetime chest X-ray exposure (any exposure) on breast cancer risk according to the number of exposures, the age at first exposure, and the first full-term pregnancy by estrogen receptor tumor status

	Estrogen receptor (ER) tumor status										
	ER negative				ER positive				Unknown		
	Controls	Cases	OR^**a**^	95% CI	Cases	OR^**a**^	95% CI	***P***^**b**^	Cases	OR^**a**^	95% CI
**Chest X-ray exposure**											
No	239	32	1		107	1			69	1	
Yes	1104	132	1.50	0.94–2.39	692	2.13	1.56–2.91	0.16	480	2.21	1.51–3.24
**Number of exposures**											
0	239	32	1		107	1			69	1	
1–3	390	43	1.31	0.77–2.25	207	1.73	1.22–2.46	0.67	142	1.87	1.21–2.87
4–9	200	29	1.99	1.10–3.62	140	2.65	1.80–3.92		82	2.51	1.55–4.07
≥10	215	21	1.51	0.79–2.90	124	2.23	1.49–3.33		118	3.27	2.03–5.28
Continuous			1.01	0.99–1.04		1.02	1.00–1.03	0.58		1.03	1.01–1.05
**Age at first exposure, years**^c^											
No exposure	239	32	0.70	0.41–1.19	107	0.53	0.38–0.75		69	0.53	0.35–0.81
≥20	490	52	1		263	1			170	1	
15–19	222	33	1.06	0.64–1.78	154	1.07	0.78–1.47	0.66	101	0.86	0.58–1.27
<15	219	27	1.13	0.66–1.94	139	1.04	0.75–1.44		124	1.25	0.86–1.82
Continuous			1.01	0.99–1.03		1.01	0.99–1.02	0.39		1.00	0.99–1.01
**According to first full-term pregnancy (FFTP)**^c^											
Only after FFTP^d^	232	35	1		141	1			92	1	
Before (incl. no FTP)	725	79	0.66	0.41–1.07	435	0.83	0.61–1.13	0.22	311	0.84	0.58–1.22

Abbreviations: OR (95% CI) odds ratio (95% confidence interval)

^**a**^Adjusted for age at censoring, birth cohort (≤1945; 1946–1959; ≥1960), number of full-term pregnancies (>2; 1–2; 0), mammography use (never; ever), educational level (intermediate/high; basic; not graduated), BMI (18.5–24.99; <18.5; ≥25 and <30; ≥30), smoking (no; current; past), and breast cancer family history (0;1st degree; 2nd degree)

^b^*P* value for χ^2^ heterogeneity test between ER-negative and ER-positive tumors

^c^Adjusted as in ^**a**^ plus number of exposures (≤5;>5)

^d^After = also includes chest X-ray exposure that occurred during the same year of first full-term pregnancy

In all analyses (Tables 4, 5, 6, and 8), there was no significant difference in the BC risk by age at first exposure, nor by timing according to the FFTP.

Because some chest X-ray variables had a high fraction of missing data, we reran the above analyses after imputing the missing data (Tables 4 and 7; Supplemental Tables 4-10 in Additional file 2.doc). The magnitude and direction of the effect estimates based on analyses using an extra class for missing data or a multiple imputation strategy were similar.

We also performed analyses restricted to cases diagnosed at most 5 years before enrollment in the study. Again, the magnitude and direction of the effect estimates were unchanged (see Supplemental Table 10 in Additional file 2.doc).

## Discussion

We found that chest X-ray exposure doubles the risk of BC in women with a hereditary predisposition to BC unexplained by a *BRCA1/2* mutation. This risk increases with the number of exposures with an increase of 3% for each additional exposure. Being born after 1960, over age 60 or a carrier of at least one variant in the DNA repair genes group associated with an increased risk of BC increased the effect of chest X-ray exposure on BC risk.

Our study confirms that low-dose ionizing radiation to the thoracic region increases the risk of BC among high-risk women, as pointed out by other studies [1, 7, 8, 18]. In contrast to Ma et al. [6] and John et al. [11], we did not find that younger age at first chest X-ray exposure was significantly associated with higher ORs compared to those initially exposed at an older age. However, we found a suggestive association between having been exposed at an early age in the subgroup of women born between 1946 and 1959 or those older than 50 years at censoring and in the subgroup of women without a family history of BC (i.e., only one sister affected for cases and none for controls) but due to the self-report exposures and potential recall bias, these results should be taken with cautious.

We also observed a difference by birth cohort on radiation-induced risk of BC, with significantly higher risks for women born after 1960, which was similar to BC risk in women carrying a *BRCA1/2* pathogenic variant and born after 1950 [3]. However, this finding was not subsequently confirmed in the Pijpe et al. study [5]. Even if radiation exposure levels were higher in the past, the decrease over the generations in the number of exposed subjects by outcome status appeared different, especially in the younger birth cohort. This may be due to the reluctance of doctors to reduce radiological examinations in women at high risk of cancer, more often classified accordingly since the discovery of the first BC predisposing genes in the 1990s [26, 27].

When stratifying on tumor ER status, we found a suggestive stronger effect of chest X-ray exposure for women with an ER^+^ tumor, consistent with Sigurdson et al.’s findings that common variants in estrogen metabolizing genes may modify the association between ionizing radiation exposure and BC risk [28].

Several strengths and weaknesses should be considered in the interpretation of our results. First, we did not include mammography in the chest X-ray exposure because we were concerned about confounding by indication, i.e., self-selection for early mammography in women with a strong family history of BC. However, all analyses were adjusted for mammography to avoid confounding. Confounding by indication for other diagnostic procedures is expected to be highly unlikely.

Potential weaknesses also include the fact that most of the cases were prevalent cases which could lead to estimates biased toward the null if radiation exposure was associated with poorer survival. Unfortunately, very little is known about the influence of exposure to ionizing radiation at any doses on overall survival and BC specific survival in high-risk BC families. Nevertheless, we performed sensitivity analyses on a subgroup of cases diagnosed within 5 years before enrollment in the study and results remained unchanged. Another potential weakness is the selection of nonrandom friends or colleagues as controls. The advantage of such controls was the greater feasibility for finding a suitable control than through a random selection in the general population, and a higher comparability for unmeasured factors with, however, the risk of sharing some risk factors with the index cases. However, friends or colleagues’ selection should not have X-ray radiation exposures related to friend or colleague relationships, and if any, BC relative risks associated with X-ray exposures would be expected to be biased towards the null. Finally, information on lifetime X-ray exposures was self-reported with accompanying potential recall biases and exposure misclassification. We relied on self-reports rather than review of medical records because of the difficulties in accessing medical records for the various diagnostic procedures. Even if methodological studies showed that the extent of misclassification was small and mainly non-differential by disease status [29, 30], an indication of relatively poorer reporting among controls, particularly for certain types of X-ray examinations and for large numbers of such examinations, was shown by Berrington et al., although it did not translate into large differences in the estimated risks [31]. Therefore, we cannot totally rule out such a bias, and results on number of exposures and age at exposures should be interpreted with caution.

One important strength of our study is that it was conducted in a homogeneous sample of high-risk women and population controls with detailed information on diagnostic procedures at different age periods. We also dealt with missing values by performing multiple imputation, which showed results with similar magnitudes and direction of effects.

Another strength was the availability of sequencing data for 113 DNA repair genes for an important subset of the study population [20]. Indeed, our study is the first to investigate the joint effect of chest X-ray exposure and rare deleterious or likely deleterious variants in DNA repair genes in women at high risk of BC. Nevertheless, we cannot exclude potential biases due to the classification of the genes according to the ORs calculated in the same population.

Moreover, we fixed a large range of ORs around 1 for the group of DNA repair genes defined as conferring no effect on BC and this might have an impact on the findings. Therefore, sensitivity analyses were performed changing the boundaries and we found similar trends in the difference in the BC risk between groups (Supplemental Table 11). We also performed a sensitivity analysis that excluded from the “High” Group the genes that were significantly (or borderline) associated with BC in our population (i.e., *ATM*, *CHEK2*, *PALB2*, *FANCM*, *MAST1*) to test whether the differential effect was driven by those genes. Again, results were unchanged (Supplemental Table 12. This analysis also pointed out, for the first time, that carriers of a rare variant in the well-established BC susceptibility genes *ATM*, *CHEK2*, or *PALB2* may be more radiosensitive than non-carriers.

Unlike previous reports, we did not find that the effect of chest X-ray exposure on BC risk was modified by family history [7, 9, 32]. This may be due to greater homogeneity in BC family history of the current sample compared with the previous studies.

## Conclusions

Our results showed that chest X-ray exposure increases BC risk 2-fold and suggested that, independent of family history, carrying rare deleterious or likely deleterious variant(s) in some DNA repair genes may modify the effect of chest X-ray exposure. Further studies are needed to evaluate other DNA repair genes or variants to identify those which could increase radiation sensitivity. Identification of sub-populations that are more or less susceptible to ionizing radiation is important and clinically relevant.

## Supplementary Information

**Additional file 1.** doc includes ‘Supplementary Method Section’ on the eligibility criteria for admission of BC patients to family cancer clinics and DNA repair-related variants identification.

**Additional file 2:** doc includes ‘Supplementary tables’. **Supplemental Table 1.** Comparison of the distribution of the characteristics between the subset of cases and controls with and without sequenced genes. **Supplemental Table 2.** Effect of lifetime chest X-ray exposure (any exposure) on breast cancer risk according to the number of exposures, the age at first exposure and the first full-term pregnancy by age at censor. **Supplemental Table 3.** Effect of lifetime chest X-ray exposure (any exposure) on breast cancer risk according to the number of exposures, the age at first exposure and the first full-term pregnancy by family history of breast cancer. **Supplemental Table 4.** Effect of variant carrier status on breast cancer in the GENESIS population. **Supplemental Table 5.** Effect of lifetime chest X-ray exposure (any exposure) on breast cancer risk according to the number of exposures, the age at first exposure and the first full-term pregnancy by birth cohort, after imputation of missing data. **Supplemental Table 6.** Effect of lifetime chest X-ray exposure (any exposure) on breast cancer risk according to the number of exposures, the age at first exposure and the first full-term pregnancy by age at censoring, after imputation of missing data. **Supplemental Table 7.** Effect of lifetime chest X-ray exposure (any exposure) on breast cancer risk according to the number of exposures, the age at first exposure and the first full-term pregnancy by family history of breast cancer and by variant carrier status, after imputation of missing data. **Supplemental Table 8.** Effect of lifetime chest X-ray exposure (any exposure) on breast cancer risk according to the number of exposures, the age at first exposure and the first full-term pregnancy stratified by variant carrier status, after imputation of missing data. **Supplemental Table 9.** Effect of lifetime chest X-ray exposure (any exposure) on breast cancer risk according to the number of exposures, the age at first exposure and the first full-term pregnancy by status of tumor estrogen receptors, after imputation of missing data. **Supplemental Table 10.** Effect of lifetime chest X-ray exposure (any exposure) on breast cancer risk according to the number of exposures, the age at first exposure and the first full-term pregnancy among cases diagnosed within 5 years before enrollment in GENESIS. **Supplemental Table 11.** Sensitivity analyses with varying bounds of OR for the definition of genetic variant group: effect of lifetime chest X-ray exposure (any exposure) on breast cancer risk according to the number of exposures, the age at first exposure and the first full-term pregnancy. **Supplemental Table 12.** Sensitivity analyses by variants group, excluding variants from the ‘High’ Group in genes individually statistically (or borderline) associated with an increased risk of breast cancer in GENESIS population.

## Data Availability

The data underlying this article will be shared on reasonable request to the corresponding author.

## Abbreviations

BC: Breast cancer
DNA: Deoxyribonucleic acid
OR: Odds ratio
CI: Confidence interval
FFTP: First full-term pregnancy
*BRCA1/2*: *BRCA1* or *BRCA2*
