# Supplementary material for: Diagnostic chest X-rays and breast cancer risk among women with a hereditary predisposition to breast cancer unexplained by a BRCA1 or BRCA2 mutation
Source: Breast Cancer Res. 2021 Aug 3;23:79. doi: 10.1186/s13058-021-01456-1 (PMC8336294; doi:10.1186/s13058-021-01456-1)
Supplement: Supplementary file 1 — Additional file 1. doc includes ‘Supplementary Method Section’ on the eligibility criteria for admission of BC patients to family cancer clinics and DNA repair-related variants identification. [file 13058_2021_1456_MOESM1_ESM.docx]

**Supplementary Methods Section**

*Eligibility criteria for admission of breast cancer patients to family cancer clinics*

The elements that would result in a family cancer clinic consultation are the: presence of several cases of breast cancer in a family branch, whether on the paternal or maternal side; early onset of cancer; multifocal and / or bilateral cancer; presence of ovarian cancer; occurrence of breast cancer in men; more rarely, pancreatic cancer, melanoma or prostate cancer

*Eligible individuals for the GENESIS study and enrollment process*

Index cases (and their affected sisters) were identified through the French family cancer clinics of the Groupe Génétique et Cancer (Unicancer) (i.e. 42 centers) and were eligible when diagnosed with infiltrating mammary or ductal adenocarcinoma, were negative for *BRCA1* and *BRCA2* mutations, and had a sister with BC. The mutation screening strategy was similar for all the clinics. Two types of controls were included: unrelated controls and unaffected sisters. The unrelated controls were selected from among the unaffected friends and/or colleagues of the cases. The year of birth of controls was matched to that of the corresponding case (+/- 3 years). The parents, brothers and unaffected sisters of the index case were also contacted, when possible. Geneticists at family cancer clinics identified index cases and invited them to participate in GENESIS by referring them to the coordinating center at Institut Curie (Paris, France). Each family cancer clinic invited index cases to participate in the study by letter or during consultations informing patients of their BRCA1/2 negative results. The coordinating center organized the inclusion of index cases and their unrelated controls. The index case then sent a response coupon to the coordinating center to obtain the complete study file including a detailed information letter and a consent form to be completed. Subjects were included in the study after they sent back their signed consent. The index case contacted her unrelated unaffected friends or colleagues and gave them an information letter and response coupon. After their agreement, the coordinating center sent them the study file including the detailed information letter and consent form. Again, unrelated subjects were included in the study once they sent back their signed consent. The coordinating center organized the collection of blood samples from the index case and other participants by sending them a prescription for blood sampling, a letter for the medical analysis laboratory or the nurse who took the blood sample, and appropriate prepaid packaging for shipment of the samples directly to the biological resource center at the Centre Léon Bérard (Lyon, France).

*DNA repair-related variants*

In our previous study that assessed the contribution of rare likely deleterious germline variants in familial BC, 113 DNA repair genes were sequenced in 1,207 cases and 1,199 population controls. Detailed information on the selection of genes and resequencing procedure is provided in Girard *et al. (1)*. The association study focused on loss-of-function variants and missense variants with a phred CADD score >20 as a predictor of pathogenicity (2), and with minor allele frequency <0.5% in GENESIS controls. For each gene, variant frequencies among cases and controls were compared. Published results per gene are shown in Table 1.

Reference List

(1) Girard E, Eon-Marchais S, Olaso R, Renault AL, Damiola F, Dondon MGet al. Familial breast cancer and DNA repair genes: Insights into known and novel susceptibility genes from the GENESIS study, and implications for multigene panel testing. Int J Cancer 2019;144(8):1962-74.

(2) Kircher M, Witten DM, Jain P, O'Roak BJ, Cooper GM, Shendure J. A general framework for estimating the relative pathogenicity of human genetic variants. Nat Genet 2014;46(3):310-5.
