# Supplementary material for: Diagnostic chest X-rays and breast cancer risk among women with a hereditary predisposition to breast cancer unexplained by a BRCA1 or BRCA2 mutation
Source: Breast Cancer Res. 2021 Aug 3;23:79. doi: 10.1186/s13058-021-01456-1 (PMC8336294; doi:10.1186/s13058-021-01456-1)
Supplement: Supplementary file 2 — Additional file 2: doc includes ‘Supplementary tables’. Supplemental Table 1. Comparison of the distribution of the characteristics between the subset of cases and controls with and without sequenced genes. Supplemental Table 2. Effect of lifetime chest X-ray exposure (any exposure) on breast cancer risk according to the number of exposures, the age at first exposure and the first full-term pregnancy by age at censor. Supplemental Table 3. Effect of lifetime chest X-ray exposure (any exposure) on breast cancer risk according to the number of exposures, the age at first exposure and the first full-term pregnancy by family history of breast cancer. Supplemental Table 4. Effect of variant carrier status on breast cancer in the GENESIS population. Supplemental Table 5. Effect of lifetime chest X-ray exposure (any exposure) on breast cancer risk according to the number of exposures, the age at first exposure and the first full-term pregnancy by birth cohort, after imputation of missing data. Supplemental Table 6. Effect of lifetime chest X-ray exposure (any exposure) on breast cancer risk according to the number of exposures, the age at first exposure and the first full-term pregnancy by age at censoring, after imputation of missing data. Supplemental Table 7. Effect of lifetime chest X-ray exposure (any exposure) on breast cancer risk according to the number of exposures, the age at first exposure and the first full-term pregnancy by family history of breast cancer and by variant carrier status, after imputation of missing data. Supplemental Table 8. Effect of lifetime chest X-ray exposure (any exposure) on breast cancer risk according to the number of exposures, the age at first exposure and the first full-term pregnancy stratified by variant carrier status, after imputation of missing data. Supplemental Table 9. Effect of lifetime chest X-ray exposure (any exposure) on breast cancer risk according to the number of exposures, the age at first exposure and the first ful [file 13058_2021_1456_MOESM2_ESM.docx]

**Supplementary tables**

**Supplemental Table 1. C**omparison of the distribution of the characteristics between the subset of cases and controls with and without sequenced genes.

**Supplemental Table 2.**

Effect of lifetime chest X-ray exposure (any exposure) on breast cancer risk according to the number of exposures, the age at first exposure and the first full-term pregnancy by age at censor.

**Supplemental Table 3.**

Effect of lifetime chest X-ray exposure (any exposure) on breast cancer risk according to the number of exposures, the age at first exposure and the first full-term pregnancy by family history of breast cancer.

**Supplemental Table 4.**

Effect of variant carrier status on breast cancer in the GENESIS population.

**Supplemental Table 5.**

Effect of lifetime chest X-ray exposure (any exposure) on breast cancer risk according to the number of exposures, the age at first exposure and the first full-term pregnancy by birth cohort, after imputation of missing data.

**Supplemental Table 6.**

Effect of lifetime chest X-ray exposure (any exposure) on breast cancer risk according to the number of exposures, the age at first exposure and the first full-term pregnancy by age at censoring, after imputation of missing data.

**Supplemental Table 7.**

Effect of lifetime chest X-ray exposure (any exposure) on breast cancer risk according to the number of exposures, the age at first exposure and the first full-term pregnancy by family history of breast cancer and by variant carrier status, after imputation of missing data.

**Supplemental Table 8.**

Effect of lifetime chest X-ray exposure (any exposure) on breast cancer risk according to the number of exposures, the age at first exposure and the first full-term pregnancy stratified by variant carrier status, after imputation of missing data.

**Supplemental Table 9.**

Effect of lifetime chest X-ray exposure (any exposure) on breast cancer risk according to the number of exposures, the age at first exposure and the first full-term pregnancy by tumor estrogen receptors status, after imputation of missing data.

**Supplemental Table 10.**

Effect of lifetime chest X-ray exposure (any exposure) on breast cancer risk according to the number of exposures, the age at first exposure and the first full-term pregnancy among cases diagnosed within 5 years of enrollment.

**Supplemental Table 11.**

Sensitivity analyses with varying bounds of ORs for the definition of genetic variant groups: effect of lifetime chest X-ray exposure (any exposure) on breast cancer risk according to the number of exposures, the age at first exposure and the first full-term pregnancy.

**Supplemental Table 12.**

Sensitivity analyses by variants group excluding variants from Group ‘High’ from genes individually statistically (or borderline) associated with an increased risk of breast cancer in the GENESIS population.

**Supplemental Table 1.** Comparison of the distribution of the characteristics between the subset of cases and controls with and without sequenced genes.

| **Characteristics** | **with sequenced genes** | **without sequenced genes** |
| --- | --- | --- |

|  | Cases  N = 997 | Controls  N = 1,162 | Cases  N = 555 | Controls  N = 201 |
| --- | --- | --- | --- | --- |
|  | No. % | No. % | No. % | No. % |
| Birth cohort |  |  |  |  |
| ≤1945 | 237 (23.8) | 261 (22.5) | 251 (45.2) | 33 (16.4) |
| 1946-59 | 543 (54.5) | 617 (53.1) | 254 (45.8) | 89 (44.3) |
| ≥1960 | 217 (21.8) | 284 (24.4) | 50 (9.0) | 79 (39.3) |
| Age at censoring, years |  |  |  |  |
| Mean (sd) | 51.7 (8.9) | 56.2 (9.5) | 47.5 (9.5) | 53.5 (11.2) |
|  |  |  |  |  |
| ≤45 | 261 (26.2) | 154 (13.3) | 252 (45.4) | 47 (23.4) |
| 46-50 | 221 (22.2) | 170 (14.6) | 115 (20.7) | 27 (13.4) |
| 51-60 | 340 (34.1) | 418 (36.0) | 133 (24.0) | 67 (33.3) |
| >60 | 175 (17.6) | 420 (36.1) | 55 (9.9) | 60 (29.9) |
| Education level |  |  |  |  |
| Intermediate/High | 521 (52.3) | 777 (66.9) | 259 (46.7) | 139 (69.2) |
| Basic | 437 (43.8) | 373 (32.1) | 277 (49.9) | 61 (30.4) |
| Not graduated | 39 (3.9) | 12 (1.0) | 19 (3.4) | 0 |
| Missing | 0 | 0 | 0 | 1 (0.5) |
| Body Mass Index |  |  |  |  |
| 18.5-24.9 | 621 (62.3) | 743 (63.9) | 398 (71.7) | 126 (62.7) |
| <18.5 | 39 (3.9) | 26 (2.2) | 30 (5.4) | 6 (3.0) |
| ≥25 and <30 | 237 (23.8) | 295 (25.4) | 104 (18.7) | 50 (24.9) |
| ≥30 | 97 (9.7) | 98 (8.4) | 23 (4.1) | 19 (9.5) |
| Missing | 3 (0.3) | 0 | 0 | 0 |
| Smoking |  |  |  |  |
| No | 511 (51.3) | 583 (50.2) | 321 (57.8) | 97 (48.3) |
| Current | 101 (10.1) | 128 (11.0) | 58 (10.5) | 30 (14.9) |
| Past | 380 (38.1) | 441 (38.0) | 170 (30.6) | 73 (36.3) |
| Missing | 5 (0.5) | 10 (0.9) | 6 (1.1) | 1 (0.5) |
| Number of full term pregnancies |  |  |  |  |
| ≥2 | 283 (28.4) | 358 (30.8) | 162 (29.2) | 53 (26.4) |
| 1-2 | 593 (59.5) | 647 (55.7) | 328 (59.1) | 116 (57.7) |
| 0 | 120 (12.0) | 156 (13.4) | 65 (11.7) | 31 (15.4) |
| Missing | 1 (0.1) | 1 (0.1) | 0 | 1 (0.5) |
| Age at first full-term pregnancy, years | |  |  |  |
| <20 | 112 (11.2) | 90 (7.8) | 67 (12.1) | 12 (6.0) |
| 20-24 | 399 (40.0) | 458 (39.4) | 229 (41.3) | 73 (36.3) |
| 25-29 | 254 (25.5) | 331 (28.5) | 139 (25.1) | 61 (30.4) |
| ≥30 | 110 (11.0) | 126 (10.8) | 55 (9.9) | 23 (11.4) |
| No full-term pregnancy | 120 (12.0) | 156 (13.4) | 65 (11.7) | 31 (15.4) |
| Missing | 2 (0.2) | 1 (0.1) | 0 | 1 (0.5) |
| Family history of breast cancer^a^ |  |  |  |  |
| None | 275 (27.6) | 823 (70.8) | 152 (27.4) | 136 (67.7) |
| 1^st^ degree | 516 (51.8) | 156 (13.4) | 302 (54.4) | 31 (15.4) |
| Only 2^nd^ degree | 206 (20.7) | 182 (15.7) | 101 (18.2) | 34 (16.9) |
| Missing | 0 | 1 (0.1) | 0 | 0 |
| Tumor estrogen receptors (ER) |  |  |  |  |
| ER+ | 604 (60.6) |  | 214 (38.6) |  |
| ER- | 123 (12.3) |  | 45 (8.1) |  |
| Missing | 270 (27.1) |  | 296 (53.3) |  |
| Chest X-ray exposure^b^ |  |  |  |  |
| Never | 135 (13.5) | 195 (16.8) | 73 (13.2) | 44 (21.9) |
| Ever | 838 (84.1) | 951 (81.8) | 466 (84.0) | 153 (76.1) |
| Missing  Number of exposures | 24 (2.4) | 16 (1.4) | 16 (2.9) | 4 (2.0) |
| 0 | 135 (13.5) | 195 (16.8) | 73 (13.2) | 44 (21.9) |
| 1-3 | 260 (26.1) | 334 (28.7) | 132 (23.8) | 56 (27.9) |
| 4-9 | 168 (16.9) | 176 (15.2) | 83 (15.0) | 24 (11.9) |
| ≥10 | 160 (16.1) | 185 (15.9) | 103 (18.6) | 30 (14.9) |
| Missing | 274 (27.5) | 272 (23.4) | 164 (29.6) | 47 (23.4) |

| Continuous | 8.0 (10.4) | 7.2 (8.8) | 9.0 (13.9) | 7.2 (8.9) |
| --- | --- | --- | --- | --- |

^a^ Excluding one affected sister per index case, "none" means no history of BC for controls or no additional BC case in the family for cases; "1^st^ degree" means 1^st^ degree family history for controls or additional 1^st^ degree relative for cases and "2^nd^ degree" means only 2^nd^ degree family history for controls or only additional 2^nd^ degree family history for cases.

**^b^** Lifetime exposures up to one year prior to diagnosis for cases and up to one year prior to date of questionnaire completion for controls

**Supplemental Table 2.** Effect of lifetime chest X-ray exposure (any exposure) on breast cancer risk according to the number of exposures, the age at first exposure and the first full-term pregnancy by age at censor.

|  | **Number of** | |  | |  | **Number of** | |  |  | **Number of** | |  |  |  |
| --- | --- | --- | --- | --- | --- | --- | --- | --- | --- | --- | --- | --- | --- | --- |
|  | **Cases** | **Ctrls** | **OR^a^** | | **95%CI** | **Cases** | **Ctrls** | **OR^a^** | **95%CI** | **Cases** | **Ctrls** | **OR^a^** | **95%CI** | **P^b^** |
|  | **Age at censor (years)** | | | | | | | | | | | | |  |
|  | **≤ 50** | | | | | **51-60** | | | | **> 60** | | | |  |
| **Chest X-ray exposure** | |  |  |  | |  |  |  |  |  |  |  |  |  |
| No | 141 | 123 | 1 |  | | 47 | 63 | 1 |  | 20 | 53 | 1 |  |  |
| Yes | 681 | 268 | 1.47 | 0.97-2.24 | | 419 | 415 | 1.33 | 0.74-2.39 | 204 | 421 | 2.31 | 1.13-4.72 | 0.48 |
| **Number of exposures** | |  |  |  | |  |  |  |  |  |  |  |  |  |
| 0 | 141 | 123 | 1 |  | | 47 | 63 | 1 |  | 20 | 53 | 1 |  |  |
| 1-3 | 208 | 122 | 1.46 | 0.90-2.36 | | 124 | 138 | 1.24 | 0.64-2.40 | 60 | 130 | 1.97 | 0.91-4.27 |  |
| 4-9 | 131 | 50 | 1.69 | 0.96-2.98 | | 72 | 79 | 1.46 | 0.72-2.95 | 48 | 71 | 3.04 | 1.31-7.02 | 0.83 |
| ≥10 | 116 | 27 | 1.29 | 0.65-2.58 | | 92 | 81 | 1.13 | 0.57-2.26 | 55 | 107 | 2.74 | 1.21-6.17 |  |
| Continuous |  |  | 1.02 | 0.98-1.05 | |  |  | 1.01 | 0.98-1.03 |  |  | 1.02 | 1.00-1.04 | 0.90 |
| **Age at first exposure, years**^c^ | |  |  |  | |  |  |  |  |  |  |  |  |  |
| No exposure | 141 | 123 | 0.67 | 0.42-1.08 | | 47 | 63 | 0.90 | 0.48-1.71 | 20 | 53 | 0.52 | 0.24-1.13 |  |
| ≥20 | 246 | 124 | 1 |  | | 153 | 188 | 1 |  | 86 | 178 | 1 |  |  |
| 15-19 | 160 | 60 | 0.89 | 0.53-1.48 | | 90 | 83 | 1.62 | 0.95-2.77 | 38 | 79 | 1.18 | 0.64-2.20 | 0.53 |
| <15 | 131 | 45 | 0.85 | 0.48-1.53 | | 101 | 66 | 1.42 | 0.83-2.42 | 58 | 108 | 1.28 | 0.74-2.22 |  |
| Continuous |  |  | 1.00 | 0.98-1.03 | |  |  | 0.99 | 0.97-1.01 |  |  | 1.00 | 0.98-1.01 | 0.75 |
| **According to first full-term pregnancy (FFTP)**^c^ | | |  |  | |  |  |  |  |  |  |  |  |  |
| Only after FFTP^d^ | 115 | 38 | 1 |  | | 91 | 99 | 1 |  | 62 | 95 | 1 |  |  |
| Before (incl. no FTP) | 443 | 197 | 0.77 | 0.44-1.34 | | 261 | 250 | 1.14 | 0.69-1.87 | 121 | 278 | 0.59 | 0.34-1.02 | 0.71 |

Abbreviations: OR (95%CI): odds ratio (95% confidence interval).

**^a^** Adjusted for age at censoring (when by birth cohort analysis) and for birth cohort (continuous) (when by age at censor analysis), number of full-term pregnancies (>2; 1-2; 0), mammography use (never; ever), educational level (intermediate/high; basic; not graduated), BMI (18.5-24.99; <18.5; ≥25 and <30; ≥30), smoking (no; current; past), and family history of breast cancer (0;1^st^ degree; 2^nd^ degree).

^b^ p-value for heterogeneity test.

^c^ Adjusted as in **^a^** plus number of exposures (≤5; >5).

^d^ After = also includes chest X-ray exposure that occurred during year of first full-term pregnancy.**Supplemental Table 3.** Effect of lifetime chest X-ray exposure (any exposure) on breast cancer risk according to the number of exposures, the age at first exposure and the first full-term pregnancy by family history of breast cancer.

|  | **Number of** | |  |  | **Number of** | |  |  | **Number of** | |  |  |  |
| --- | --- | --- | --- | --- | --- | --- | --- | --- | --- | --- | --- | --- | --- |
|  | **Cases** | **Ctrls** | **OR^a^** | **95%CI** | **Cases** | **Ctrls** | **OR^a^** | **95%CI** | **Cases** | **Ctrls** | **OR^a^** | **95%CI** | **P^b^** |
|  | **Family history of breast cancer** | | | | | | | | | | | |  |
|  | **None^c^** | | | | **1^st^ degree^c^** | | | | **Only 2^nd^ degree^c^** | | | |  |
| **Chest X-ray exposure** |  |  |  |  |  |  |  |  |  |  |  |  |  |
| No | 57 | 153 | 1 |  | 106 | 39 | 1 |  | 45 | 47 | 1 |  |  |
| Yes | 359 | 793 | 2.22 | 1.44-3.44 | 688 | 145 | 2.08 | 1.26-3.43 | 257 | 165 | 1.81 | 1.01-3.26 | 0.76 |
| **Number of exposures** |  |  |  |  |  |  |  |  |  |  |  |  |  |
| 0 | 57 | 153 | 1 |  | 106 | 39 | 1 |  | 45 | 47 | 1 |  |  |
| 1-3 | 107 | 274 | 1.96 | 1.20-3.18 | 205 | 48 | 1.73 | 0.96-3.09 | 80 | 67 | 1.32 | 0.68-2.56 |  |
| 4-9 | 73 | 142 | 2.69 | 1.57-4.61 | 119 | 27 | 2.10 | 1.08-4.06 | 59 | 31 | 2.83 | 1.33-6.00 | 0.38 |
| ≥10 | 73 | 155 | 2.95 | 1.71-5.11 | 143 | 34 | 1.87 | 0.98-3.58 | 47 | 26 | 2.12 | 0.92-4.90 |  |
| Continuous |  |  | 1.02 | 1.01-1.04 |  |  | 1.04 | 1.01-1.07 |  |  | 1.02 | 0.99-1.05 | 0.47 |
| **Age at first exposure, years**^d^ | |  |  |  |  |  |  |  |  |  |  |  |  |
| No exposure | 57 | 153 | 0.54 | 0.34-0.88 | 106 | 39 | 0.55 | 0.31-0.97 | 45 | 47 | 0.55 | 0.29-1.06 |  |
| ≥20 | 133 | 362 | 1 |  | 248 | 60 | 1 |  | 104 | 68 | 1 |  |  |
| 15-19 | 76 | 158 | 1.08 | 0.71-1.63 | 150 | 23 | 1.11 | 0.60-2.05 | 62 | 41 | 0.85 | 0.44-1.64 | 0.63 |
| <15 | 85 | 147 | 1.48 | 0.97-2.24 | 154 | 36 | 0.99 | 0.57-1.71 | 51 | 35 | 0.69 | 0.35-1.36 |  |
| Continuous |  |  | 0.99 | 0.98-1.01 |  |  | 1.01 | 0.99-1.04 |  |  | 1.02 | 0.99-1.05 | 0.15 |
| **According to first full-term pregnancy (FFTP)**^d^ | | |  |  |  |  |  |  |  |  |  |  |  |
| Only after FFTP^e^ | 76 | 181 | 1 |  | 134 | 23 | 1 |  | 58 | 28 | 1 |  |  |
| Before (incl. no FTP) | 223 | 505 | 0.93 | 0.63-1.38 | 439 | 102 | 0.53 | 0.30-0.95 | 163 | 117 | 0.93 | 0.49-1.77 | 0.29 |

Abbreviations: OR (95%CI): odds ratio (95% confidence interval).

**^a^** Adjusted for age at censoring, birth cohort (≤1945; 1946-1959; ≥1960), number of full-term pregnancies (>2; 1-2; 0), mammography use (never; ever), educational level (intermediate/high; basic; not graduated), BMI (18.5-24.99; <18.5; ≥25 and <30; ≥30), smoking (no; current; past).

^b^ p-value for heterogeneity test.

^c^ “None” means no history of BC for controls or no additional BC case in the family for cases; "1^st^ degree" means 1^st^ degree family history for controls or additional 1^st^ degree relative for cases and "2^nd^ degree" means only 2^nd^ degree family history for controls or only additional 2nd degree family history for cases

^d^ Adjusted as in **^a^** plus number of exposures (≤5;>5).

^e^ After = also includes chest X-ray exposure that occurred during the same year of first full-term pregnancy.

**Supplemental Table 4.** Effect of variant carrier status on breast cancer in the GENESIS population (20).

| **DNA repair rare variants** |  | | | | **Multiple imputation**  **for the adjustment variables** | |
| --- | --- | --- | --- | --- | --- | --- |
|  | **Number of** | |  |  |  |  |
|  | **Cases** | **Ctrls** | **OR^a^** | **95%CI** | **OR^a^** | **95%CI** |
| **‘Low’^b^ Group** |  |  |  |  |  |  |
| No | 723 | 721 | 1 |  | 1 |  |
| Yes | 274 | 441 | 0.62 | 0.50-0.77 | 0.62 | 0.50-0.77 |
| **Number of variants** |  |  |  |  |  |  |
| 0 | 723 | 721 | 1 |  | 1 |  |
| 1 | 227 | 341 | 0.66 | 0.53-0.84 | 0.67 | 0.53-0.84 |
| 2 | 44 | 85 | 0.52 | 0.34-0.81 | 0.51 | 0.33-0.79 |
| $\geq$3 | 3 | 15 | 0.26 | 0.07-1.03 | 0.25 | 0.06-1.01 |
| Continuous |  |  | 0.69 | 0.59-0.81 | 0.69 | 0.59-0.81 |
| **‘No Effect’ Group ^c^** |  |  |  |  |  |  |
| No | 575 | 659 | 1 |  | 1 |  |
| Yes | 422 | 503 | 1.02 | 0.84-1.25 | 1.03 | 0.84-1.25 |
| **Number of variants** |  |  |  |  |  |  |
| 0 | 575 | 659 | 1 |  | 1 |  |
| 1 | 309 | 389 | 0.97 | 0.78-1.21 | 0.97 | 0.78-1.21 |
| 2 | 96 | 92 | 1.16 | 0.81-1.67 | 1.17 | 0.82-1.67 |
| $\geq$3 | 17 | 22 | 1.32 | 0.62-2.80 | 1.32 | 0.62-2.77 |
| Continuous |  |  | 1.07 | 0.93-1.22 | 1.06 | 0.93-1.22 |
| **‘High’Group ^d^** |  |  |  |  |  |  |
| No | 422 | 667 | 1 |  | 1 |  |
| Yes | 575 | 495 | 1.98 | 1.62-2.42 | 1.98 | 1.62-2.42 |
| **Number of variants** |  |  |  |  |  |  |
| 0 | 422 | 667 | 1 |  | 1 |  |
| 1 | 381 | 357 | 1.86 | 1.49-2.32 | 1.81 | 1.45-2.26 |
| 2 | 142 | 116 | 1.99 | 1.44-2.74 | 1.95 | 1.42-2.68 |
| $\geq$3 | 52 | 22 | 3.91 | 2.15-7.08 | 3.88 | 2.15-7.01 |
| Continuous |  |  | 1.52 | 1.34-1.71 | 1.51 | 1.34-1.71 |

Abbreviations: OR (95%CI): odds ratio (95% confidence interval).

**^a^** Adjusted for age at censoring, birth cohort (≤1945; 1946-1959; ≥1960), number of full-term pregnancies (>2; 1-2; 0), mammography use (never; ever), educational level (intermediate/high; basic; not graduated), BMI (18.5-24.99; <18.5; ≥25 and <30; ≥30), smoking (no; current; past), and other DNA repair genes groups.

**^b^** at least one variant in a gene from the ‘Low’ Group; **^c^** at least one variant in a gene from the ‘No Effect’ Group; **^d^** at least one variant in a gene from the ‘High’ Group.

**Supplemental Table 5.** Effect of lifetime chest X-ray exposure (any exposure) on breast cancer risk according to the number of exposures, the age at first exposure and the first full-term pregnancy by birth cohort, after imputation of missing data.

|  | **Birth cohort** | | | | | | | | | | | |
| --- | --- | --- | --- | --- | --- | --- | --- | --- | --- | --- | --- | --- |
|  | **≤1945** | | | | **1946-1959** | | | | **≥1960** | | | |
|  | **Number of** | |  |  | **Number of** | |  |  | **Number of** | |  |  |
|  | **Cases** | **Ctrls** | **OR^a^** | **95%CI** | **Cases** | **Ctrls** | **OR**^a^ | **95%CI** | **Cases** | **Ctrls** | **OR**^a^ | **95%CI** |
| **Chest X-ray exposure** |  |  |  |  |  |  |  |  |  |  |  |  |
| No | 47 | 31 | 1 |  | 96 | 92 | 1 |  | 65 | 116 | 1 |  |
| Yes | 431 | 259 | 1.74 | 0.79-3.81 | 678 | 605 | 1.59 | 1.03-2.47 | 195 | 240 | 2.54 | 1.62-3.99 |
| **Number of exposures** |  |  |  |  |  |  |  |  |  |  |  |  |
| 0 | 47 | 31 | 1 |  | 96 | 92 | 1 |  | 65 | 116 | 1 |  |
| 1-3 | 134 | 81 | 1.84 | 0.79-4.31 | 179 | 197 | 1.13 | 0.68-1.85 | 79 | 112 | 1.97 | 1.18-3.27 |
| 4-9 | 81 | 46 | 1.63 | 0.68-3.92 | 122 | 111 | 1.61 | 0.98-2.63 | 48 | 43 | 3.47 | 2.00-6.02 |
| ≥10 | 105 | 64 | 1.75 | 0.74-4.13 | 142 | 130 | 2.58 | 1.51-4.39 | 16 | 21 | 3.11 | 1.36-7.12 |
| Continuous |  |  | 1.02 | 0.99-1.04 |  |  | 1.05 | 1.03-1.07 |  |  | 1.08 | 1.03-1.13 |
| **Age at first exposure, years**^b^ | |  |  |  |  |  |  |  |  |  |  |  |
| No exposure | 47 | 31 | 0.48 | 0.20-1.13 | 96 | 92 | 0.90 | 0.55-1.46 | 65 | 116 | 0.45 | 0.27-0.74 |
| ≥20 | 163 | 119 | 1 |  | 237 | 261 | 1 |  | 85 | 110 | 1 |  |
| 15-19 | 87 | 45 | 0.80 | 0.42-1.52 | 159 | 123 | 0.99 | 0.64-1.52 | 42 | 54 | 1.11 | 0.63-1.95 |
| <15 | 122 | 64 | 0.89 | 0.51-1.55 | 140 | 114 | 1.25 | 0.81-1.94 | 28 | 41 | 0.89 | 0.45-1.73 |
| Continuous |  |  | 1.00 | 0.99-1.02 |  |  | 1.00 | 0.99-1.02 |  |  | 1.02 | 0.99-1.05 |
| **According to 1^st^ full-term pregnancy (FFTP)**^b^ | | |  |  |  |  |  |  |  |  |  |  |
| Only after FFTP**^c^** | 109 | 59 | 1 |  | 124 | 139 | 1 |  | 35 | 34 | 1 |  |
| Before (incl. no FTP) | 268 | 175 | 0.60 | 0.34-1.07 | 429 | 373 | 0.94 | 0.63-1.42 | 128 | 177 | 0.84 | 0.44-1.57 |

Abbreviations: OR (95%CI): odds ratio (95% confidence interval)

^a^ Adjusted for age at censoring, no. of first full-term pregnancies (>2; 1-2; 0), mammography use (never; ever), educational level (intermediate/high; basic; not graduated), BMI (18.5-24.9; <18.5; ≥25 and <30; ≥30), smoking (no; current; past), and breast cancer family history (0; 1^st^ degree; 2^nd^ degree).

^b^ Adjusted as in **^a^** plus number of exposures (≤5; >5).

**^c^** After = also includes chest X-ray exposure that occurred during the same year of first full-term pregnancy.**Supplemental Table 6.** Effect of lifetime chest X-ray exposure (any exposure) on breast cancer risk according to the number of exposures, the age at first exposure and the first full-term pregnancy by age at censoring, after imputation of missing data.

|  | **Age at censor (years)** | | | | | | | | | | | |
| --- | --- | --- | --- | --- | --- | --- | --- | --- | --- | --- | --- | --- |
|  | **≤ 50** | | | | **51-60** | | | | **> 60** | | | |
|  | **Number of** | |  |  | **Number of** | |  |  | **Number of** | |  |  |
|  | **Cases** | **Ctrls** | **OR^a^** | **95%CI** | **Cases** | **Ctrls** | **OR^a^** | **95%CI** | **Cases** | **Ctrols** | **OR^a^** | **95%CI** |
| **Chest X-ray exposure** |  |  |  |  |  |  |  |  |  |  |  |  |
| No | 141 | 123 | 1 |  | 47 | 63 | 1 |  | 20 | 53 | 1 |  |
| Yes | 681 | 268 | 1.50 | 0.99-2.27 | 419 | 415 | 1.30 | 0.73-2.34 | 204 | 421 | 2.36 | 1.15-4.86 |
| **Number of exposures** |  |  |  |  |  |  |  |  |  |  |  |  |
| 0 | 141 | 123 | 1 |  | 47 | 63 | 1 |  | 20 | 53 | 1 |  |
| 1-3 | 208 | 122 | 1.38 | 0.86-2.21 | 124 | 138 | 1.16 | 1.60-2.23 | 60 | 130 | 2.02 | 0.93-4.40 |
| 4-9 | 131 | 50 | 1.67 | 1.01-2.75 | 72 | 79 | 1.43 | 0.75-2.72 | 48 | 71 | 2.58 | 1.15-5.80 |
| ≥10 | 116 | 27 | 1.51 | 0.77-2.96 | 92 | 81 | 1.35 | 0.69-2.64 | 55 | 107 | 2.64 | 1.19-5.87 |
| Continuous |  |  | 1.02 | 0.99-1.06 |  |  | 1.01 | 0.99-1.04 |  |  | 1.02 | ]1.00-1.05 |
| **Age at first exposure, years**^b^ | |  |  |  |  |  |  |  |  |  |  |  |
| No exposure | 141 | 123 | 0.67 | 0.42-1.07 | 47 | 63 | 1.04 | 0.55-1.96 | 20 | 53 | 0.48 | 0.22-1.05 |
| ≥20 | 246 | 124 | 1 |  | 153 | 188 | 1 |  | 86 | 178 | 1 |  |
| 15-19 | 160 | 60 | 0.94 | 0.56-1.58 | 90 | 83 | 1.38 | 0.81-2.34 | 38 | 79 | 1.22 | 0.66-2.26 |
| <15 | 131 | 45 | 0.86 | 0.48-1.56 | 101 | 66 | 1.30 | 0.77-2.20 | 58 | 108 | 1.29 | 0.75-2.20 |
| Continuous |  |  | 1.00 | 0.98-1.03 |  |  | 1.00 | 0.98-1.02 |  |  | 1.00 | 0.98-1.01 |
| **According to 1^st^ full-term pregnancy (FFTP)**^b^ | | |  |  |  |  |  |  |  |  |  |  |
| Only after FFTP**^c^** | 115 | 38 | 1 |  | 91 | 99 | 1 |  | 62 | 95 | 1 |  |
| Before (incl. no FTP) | 443 | 197 | 0.82 | 0.45-1.41 | 261 | 250 | 1.08 | 0.66-1.77 | 121 | 278 | 0.69 | 0.41-1.18 |

Abbreviations: OR (95%CI): odds ratio (95% confidence interval).

**^a^** Adjusted for year of birth (continuous), no. of first full-term pregnancies (>2; 1-2; 0), mammography use (never; ever), educational level (intermediate/high; basic; not graduated), BMI (18.5-24.99; <18.5; ≥25 and <30; ≥30), smoking (no; current; past), and breast cancer family history (0; 1^st^ degree; 2^nd^ degree).

^b^ Adjusted as in **^a^** plus number of exposures (≤5; >5).

**^c^** After = also includes chest X-ray exposure that occurred during the same year of first full-term pregnancy.

**Supplemental Table 7.** Effect of lifetime chest X-ray exposure (any exposure) on breast cancer risk according to the number of exposures, the age at first exposure and the first full-term pregnancy by family history of breast cancer and by variant carrier status, after imputation of missing data.

|  | **Family history of breast cancer** | | | | | | | | | | | |
| --- | --- | --- | --- | --- | --- | --- | --- | --- | --- | --- | --- | --- |
|  | **None^a^** | | | | **1^st^ degree^a^** | | | | **Only 2^nd^ degree^a^** | | | |
|  | **Number of** | |  |  | **Number of** | |  |  | **Number of** | |  |  |
|  | **Cases** | **Ctrls** | **OR^b^** | **95%CI** | **Cases** | **Ctrls** | **OR^b^** | **95%CI** | **Cases** |  | **OR^b^** | **95%CI** |
| **Chest X-ray exposure** |  |  |  |  |  |  |  |  |  |  |  |  |
| No | 57 | 153 | 1 |  | 106 | 39 | 1 |  | 45 | 47 | 1 |  |
| Yes | 359 | 793 | 2.18 | 1.41-3.36 | 688 | 145 | 2.09 | 1.26-3.44 | 257 | 165 | 1.81 | 1.01-3.25 |
| **Number of exposures** |  |  |  |  |  |  |  |  |  |  |  |  |
| 0 | 57 | 153 | 1 |  | 106 | 39 | 1 |  | 45 | 47 | 1 |  |
| 1-3 | 107 | 274 | 1.81 | 1.12-2.93 | 205 | 48 | 1.65 | 0.93-2.94 | 80 | 67 | 1.32 | 0.68-2.54 |
| 4-9 | 73 | 142 | 2.27 | 1.39-3.70 | 119 | 27 | 2.29 | 1.27-4.13 | 59 | 31 | 2.44 | 1.23-4.56 |
| ≥10 | 73 | 155 | 3.00 | 1.77-5.10 | 143 | 34 | 2.65 | 1.38-5.09 | 47 | 26 | 2.35 | 1.05-5.24 |
| Continuous |  |  | 1.03 | 1.01-1.05 |  |  | 1.05 | 1.02-1.09 |  |  | 1.02 | 0.99-1.05 |
| **Age at first exposure, years**^c^ | |  |  |  |  |  |  |  |  |  |  |  |
| No exposure | 57 | 153 | 0.57 | 0.35-0.91 | 106 | 39 | 0.58 | 0.33-1.02 | 45 | 47 | 0.60 | 0.31-1.15 |
| ≥20 | 133 | 362 | 1 |  | 248 | 60 | 1 |  | 104 | 68 | 1 |  |
| 15-19 | 76 | 158 | 1.11 | 0.74-1.68 | 150 | 23 | 0.96 | 0.53-1.73 | 62 | 41 | 0.89 | 0.47-1.69 |
| <15 | 85 | 147 | 1.45 | 0.96-2.20 | 154 | 36 | 0.86 | 0.50-1.48 | 51 | 35 | 0.72 | 0.37-1.41 |
| Continuous |  |  | 1.00 | 0.98-1.01 |  |  | 1.02 | 1.00-1.04 |  |  |  |  |
| **According to 1^st^ full-term pregnancy (FFTP)**^c^ | | |  |  |  |  |  |  |  |  |  |  |
| Only after FFTP^d^ | 76 | 181 | 1 |  | 134 | 23 | 1 |  | 58 | 28 | 1 |  |
| Before (incl. no FTP) | 223 | 505 | 0.98 | 0.66-1.45 | 439 | 102 | 0.55 | 0.31-0.98 | 163 | 117 | 0.98 | 0.52-1.85 |

Abbreviations: OR (95%CI): odds ratio (95% confidence interval).

**^a^** “None” means no history of BC for controls or no additional BC case in the family for cases; "1^st^ degree" means 1^st^ degree family history for controls or additional 1^st^ degree relative for cases and "2^nd^ degree" means only 2^nd^ degree family history for controls or only additional 2nd degree family history for cases

**^b^** Adjusted for age at censoring, birth cohort (≤1945; 1946-1959; ≥1960), number of full-term pregnancies (>2; 1-2; 0), mammography use (never; ever), educational level (intermediate/high; basic; not graduated), BMI (18.5-24.99; <18.5; ≥25 and <30; ≥30), and smoking (no; current; past).

^c^ Adjusted as in **^b^** plus number of exposures (≤5; >5).

^d^ After = also includes chest X-ray exposure that occurred during the same year of first full-term pregnancy.

**Supplemental Table 8.** Effect of lifetime chest X-ray exposure (any exposure) on breast cancer risk according to the number of exposures, the age at first exposure and the first full-term pregnancy stratified by variant carrier status, after imputation of missing data.

|  | **Rare DNA repair variant** | | | | | | | | | | | |
| --- | --- | --- | --- | --- | --- | --- | --- | --- | --- | --- | --- | --- |
|  | **’Low’ Group^d^** | | | | **‘No Effect’ Group ^e^** | | | | **‘High’ Group ^f^** | | | |
|  | **Number of** | |  |  | **Number of** | |  |  | **Number of** | |  |  |
|  | **Cases** | **Ctrls** | **OR^a^** | **95%CI** | **Cases** | **Ctrls** | **OR^a^** | **95%CI** | **Cases** |  | **OR^a^** | **95%CI** |
| **Chest X-ray exposure** |  |  |  |  |  |  |  |  |  |  |  |  |
| No | 34 | 67 | 1 |  | 61 | 81 | 1 |  | 65 | 91 | 1 |  |
| Yes | 234 | 371 | 1.96 | 1.13-3.42 | 354 | 416 | 1.63 | 1.03-2.58 | 495 | 400 | 3.26 | 2.11-5.03 |
| **Number of exposures** |  |  |  |  |  |  |  |  |  |  |  |  |
| 0 | 34 | 67 | 1 |  | 61 | 81 | 1 |  | 65 | 91 | 1 |  |
| <10 | 119 | 183 | 1.86 | 1.06-3.28 | 184 | 215 | 1.53 | 0.96-2.44 | 256 | 219 | 3.04 | 1.95-4.74 |
| ≥10 | 47 | 75 | 2.40 | 1.21-4.78 | 67 | 85 | 2.20 | 1.24-3.90 | 94 | 78 | 4.27 | 2.48-7.34 |
| Continuous |  |  | 1.02 | 0.99-1.05 |  |  | 1.03 | 1.01-1.05 |  |  | 1.05 | 1.03-1.07 |
| **Age at first exposure, years**^b^ | |  |  |  |  |  |  |  |  |  |  |  |
| No exposure | 34 | 67 | 0.60 | 0.32-1.10 | 61 | 81 | 0.82 | 0.50-1.35 | 65 | 91 | 0.38 | 0.24-0.61 |
| ≥20 | 90 | 163 | 1 |  | 124 | 188 | 1 |  | 179 | 180 | 1 |  |
| <20 | 101 | 143 | 1.25 | 0.81-1.92 | 166 | 165 | 1.34 | 0.92-1.94 | 222 | 159 | 1.20 | 0.85-1.70 |
| Continuous |  |  | 1.00 | 0.98-1.02 |  |  | 1.00 | 0.98-1.01 |  |  | 1.00 | 0.98-1.01 |
| **According to 1^st^ full-term pregnancy (FFTP)**^b^ | | |  |  |  |  |  |  |  |  |  |  |
| Only after FFTP**^c^** | 52 | 75 | 1 |  | 68 | 91 | 1 |  | 95 | 92 | 1 |  |
| Before (incl. no FTP) | 142 | 242 | 0.89 | 0.54-1.49 | 231 | 271 | 0.93 | 0.60-1.44 | 311 | 257 | 1.03 | 0.69-1.56 |

Abbreviations: OR (95%CI): odds ratio (95% confidence interval)

**^a^** Adjusted for age at censoring, birth cohort (≤1945; 1946-1959; ≥1960), number of full-term pregnancies (>2; 1-2; 0), mammography use (never; ever), educational level (intermediate/high; basic; not graduated), BMI (18.5-24.9; <18.5; ≥25 and <30; ≥30), smoking (no; current; past), and both other DNA repair genes groups.

^b^ Adjusted as in **^a^** plus number of exposures (≤5;>5).

**^c^** After = also includes chest X-ray exposure that occurred during the same year of first full-term pregnancy.

**^d^** at least one variant in a gene from the ‘Low’ Group; **^e^** at least one variant in a gene from the ‘No Effect’ Group; **^f^** at least one variant in a gene from the ‘High’ Group.

**Supplemental Table 9.** Effect of lifetime chest X-ray exposure (any exposure) on breast cancer risk according to the number of exposures, the age at first exposure and the first full-term pregnancy by status of tumor estrogen receptors status, after imputation of missing data.

|  | **Estrogen receptor (ER) tumor status** | | | | | | | | | |
| --- | --- | --- | --- | --- | --- | --- | --- | --- | --- | --- |
|  | **ER negative** | | | | **ER positive** | | | **Unknown ER status** | | |
|  | **Ctrls** | **Cases** | **OR^a^** | **95%CI** | **Cases** | **OR^a^** | **95%CI** | **Cases** | **OR^a^** | **95%CI** |
| **Chest X-ray exposure** |  |  |  |  |  |  |  |  |  |  |
| No | 239 | 32 | 1 |  | 107 | 1 |  | 69 | 1 |  |
| Yes | 1,104 | 132 | 1.51 | 0.95-2.41 | 692 | 2.12 | 1.55-2.90 | 480 | 2.19 | 1.50-3.22 |
| **Number of exposures** |  |  |  |  |  |  |  |  |  |  |
| 0 | 239 | 32 | 1 |  | 107 | 1 |  | 69 | 1 |  |
| 1-3 | 390 | 43 | 1.24 | 0.73-2.11 | 207 | 1.67 | 1.18-2.37 | 142 | 1.73 | 1.13-2.65 |
| 4-9 | 200 | 29 | 1.65 | 0.96-2.83 | 140 | 2.45 | 1.73-3.48 | 82 | 2.26 | 1.43-3.49 |
| ≥10 | 215 | 21 | 2.01 | 1.09-3.71 | 124 | 2.64 | 1.78-3.91 | 118 | 3.26 | 2.04-5.20 |
| Continuous |  |  | 1.03 | 1.00-1.05 |  | 1.03 | 1.02-1.05 | 155 | 1.04 | 1.03-1.06 |
| **Age at first exposure, years**^b^ |  |  |  |  |  |  |  |  |  |  |
| No exposure | 239 | 32 | 0.78 | 0.46-1.33 | 107 | 0.55 | 0.39-0.78 | 69 | 0.56 | 0.37-0.86 |
| ≥20 | 490 | 52 | 1 |  | 263 | 1 |  | 170 | 1 |  |
| 15-19 | 222 | 33 | 1.04 | 0.62-1.75 | 154 | 1.04 | 0.76-1.43 | 101 | 0.91 | 0.62-1.33 |
| <15 | 219 | 27 | 1.12 | 0.65-1.91 | 139 | 1.00 | 0.72-1.37 | 124 | 1.25 | 0.86-1.80 |
| Continuous |  |  | 1.01 | 0.99-1.03 |  | 1.01 | 0.99-1.02 |  | 1.00 | 0.99-1.02 |
| **According to 1^st^ full-term pregnancy (FFTP)**^b^ | | |  |  |  |  |  |  |  |  |
| Only after FFTP**^c^** | 232 | 35 | 1 |  | 141 | 1 |  | 92 | 1 |  |
| Before (incl. no FTP) | 725 | 79 | 0.72 | 0.44-1.17 | 435 | 0.86 | 0.63-1.15 | 311 | 0.91 | 0.64-1.31 |

Abbreviations: OR (95%CI): odds ratio (95% confidence interval).

**^a^** Adjusted for age at censoring, birth cohort (≤1945; 1946-1959; ≥1960), number of full-term pregnancies (>2; 1-2; 0), mammography use (never; ever), educational level (intermediate/high; basic; not graduated), BMI (18.5-24.9; <18.5; ≥25 and <30; ≥30), smoking (no; current; past), and breast cancer family history (0;1^st^ degree; 2^nd^ degree).

^b^ Adjusted as in **^a^** plus number of exposures (≤5;>5).

**^c^** After = also includes chest X-ray exposure that occurred during the same year of first full-term pregnancy.

**Supplemental Table 10.** Effect of lifetime chest X-ray exposure (any exposure) on breast cancer risk according to the number of exposures, the age at first exposure and the first full-term pregnancy among cases diagnosed within 5 years of enrollment

|  | **Number of** | | **OR**^a^ | **95%CI** | **Multiple imputation** | |
| --- | --- | --- | --- | --- | --- | --- |
|  | **Cases** | **Controls** |  |  | **OR**^a^ | **95%CI** |
| **Chest X-ray exposure** |  |  |  |  |  |  |
| Never | 92 | 239 | 1 |  | 1 |  |
| Ever | 589 | 1,104 | 2.18 | 1.57-3.03 | 2.17 | 1.56-3.01 |
| **Number of exposures** |  |  |  |  |  |  |
| 0 | 92 | 239 | 1 |  | 1 |  |
| 1-3 | 194 | 390 | 1.91 | 1.33-2.75 | 1.82 | 1.27-2.62 |
| 4-9 | 124 | 200 | 2.70 | 1.80-4.06 | 2.46 | 1.70-3.56 |
| ≥10 | 111 | 215 | 2.30 | 1.51-3.51 | 2.55 | 1.69-3.86 |
| Continuous |  |  | 1.02 | 1.00-1.03 | 1.03 | 1.01-1.04 |
| **Age at first exposure, years**^b^ |  |  |  |  |  |  |
| No exposure | 92 | 239 | 0.51 | 0.35-0.73 | 0.53 | 0.37-0.75 |
| ≥20 | 234 | 490 | 1 |  | 1 |  |
| 15-19 | 129 | 222 | 1.07 | 0.77-1.48 | 1.05 | 0.76-1.45 |
| <15 | 121 | 219 | 1.07 | 0.77-1.48 | 1.03 | 0.75-1.43 |
| Continuous |  |  | 1.01 | 1.00-1.02 | 1.01 | 0.99-1.02 |
| **According to 1^st^ full-term pregnancy (FFTP)**^b^ | | |  |  |  |  |
| Only after FFTP^c^ | 134 | 232 | 1 |  | 1 |  |
| Before (incl. no FTP) | 363 | 725 | 0.76 | 0.56-1.04 | 0.80 | 0.59-1.08 |
| **According to 1^st^ FFTP & lifetime number** | | |  |  |  |  |
| Only after & ≤5 | 95 | 178 | 1 |  | 1 |  |
| Only after & >5 | 29 | 43 | 1.96 | 1.04-3.68 | 1.79 | 1.02-3.15 |
| Before & ≤5 (incl. no FTP) | 218 | 412 | 0.98 | 0.66-1.44 | 0.90 | 0.62-1.31 |
| Before & >5 (incl. no FTP) | 109 | 215 | 0.99 | 0.66-1.50 | 1.17 | 0.81-1.69 |

Abbreviations: OR (95%CI): odds ratio (95% confidence interval).

**^a^** Adjusted for age at censoring, birth cohort (≤1945; 1946-1959; ≥1960), number of full-term pregnancies (>2; 1-2; 0), mammography use (never; ever), educational level (intermediate/high; basic; not graduated), BMI (18.5-24.99; <18.5; ≥25 and <30; ≥30), smoking (no; current; past), and breast cancer family history (0;1^st^ degree; 2^nd^ degree).

^b^ Adjusted as in **^a^** plus number of exposures (≤5; >5).

^c^ After = also includes chest X-ray exposure that occurred during the same year of first full-term pregnancy.

**Supplemental Table 11.** Sensitivity analyses with varying bounds of ORs for the definition of genetic variant group: effect of lifetime chest X-ray exposure (any exposure) on breast cancer risk according to the number of exposures, the age at first exposure and the first full-term pregnancy.

|  | **Rare DNA repair variants** | | | | | | | | | | | |  |
| --- | --- | --- | --- | --- | --- | --- | --- | --- | --- | --- | --- | --- | --- |
|  | **‘Low’ Group ^e^** | | | | **‘No Effect’ Group ^f^** | | | | **‘High’ Group ^g^** | | | |  |
|  | **Number of** | |  |  | **Number of** | |  |  | **Number of** | |  |  |  |
|  | **Cases** | **Ctrls** | **OR^a^** | **95%CI** | **Cases** | **Ctrls** | **OR^a^** | **95%CI** | **Cases** | **Ctrls** | **OR^a^** | **95%CI** | **P^b^** |
|  | **OR<0.8** | | | | **0.8≤OR≤1.2** | | | | **OR>1.2** | | | |  |
| **Chest X-ray exposure** |  |  |  |  |  |  |  |  |  |  |  |  |  |
| No | 16 | 44 | 1 |  | 84 | 122 | 1 |  | 47 | 66 | 1 |  |  |
| Yes | 117 | 228 | 2.62 | 1.20-5.71 | 511 | 597 | 1.79 | 1.23-2.60 | 412 | 287 | 3.82 | 2.28-6.42 | 0.07 |
| **Number of exposures** |  |  |  |  |  |  |  |  |  |  |  |  |  |
| 0 | 16 | 44 | 1 |  | 84 | 122 | 1 |  | 47 | 66 | 1 |  |  |
| <10 | 55 | 117 | 2.09 | 0.91-4.76 | 267 | 317 | 1.69 | 1.14-2.51 | 211 | 157 | 3.45 | 2.01-5.92 | 0.06 |
| ≥10 | 27 | 43 | 4.48 | 1.66-12.1 | 97 | 115 | 2.17 | 1.34-3.53 | 77 | 59 | 4.24 | 2.22-8.12 |  |
| Continuous |  |  | 1.04 | 1.00-1.07 |  |  | 1.03 | 1.01-1.05 |  |  | 1.04 | 1.02-1.06 | 0.8 |
| **Age at first exposure, years**^d^ | |  |  |  |  |  |  |  |  |  |  |  |  |
| No exposure | 16 | 44 | 0.51 | 0.22-1.22 | 84 | 122 | 0.73 | 0.49-1.11 | 47 | 66 | 0.33 | 0.19-0.57 |  |
| ≥20 | 48 | 108 | 1 |  | 181 | 272 | 1 |  | 150 | 131 | 1 |  | 0.7 |
| <20 | 47 | 84 | 1.29 | 0.71-2.34 | 238 | 236 | 1.37 | 1.00-1.88 | 182 | 118 | 1.22 | 0.82-1.82 |  |
| Continuous |  |  | 1.01 | 0.98-1.04 |  |  | 0.99 | 0.98-1.01 |  |  | 1.00 | 0.98-1.02 | 0.3 |
| **According to first full-term pregnancy (FFTP)**^d^ | | |  |  |  |  |  |  |  |  |  |  |  |
| Only after FFTP ^e^ | 28 | 45 | 1 |  | 101 | 134 | 1 |  | 74 | 68 | 1 |  |  |
| Before (incl. no FTP) | 69 | 151 | 0.66 | 0.33-1.34 | 327 | 389 | 0.96 | 0.67-1.39 | 263 | 189 | 1.15 | 0.71-1.86 | 0.6 |
|  | **OR<1.0** | | | | **OR=1.0** | | | | **OR>1.0** | | | |  |
| **Chest X-ray exposure** |  |  |  |  |  |  |  |  |  |  |  |  |  |
| No | 48 | 86 | 1 |  | 27 | 37 | 1 |  | 78 | 106 | 1 |  |  |
| Yes | 306 | 436 | 2.01 | 1.26-3.22 | 182 | 204 | 1.61 | 0.84-3.10 | 561 | 491 | 2.75 | 1.85-4.07 | 0.03 |
| **Number of exposures** |  |  |  |  |  |  |  |  |  |  |  |  |  |
| 0 | 48 | 86 | 1 |  | 27 | 37 | 1 |  | 78 | 106 | 1 |  |  |
| <10 | 157 | 221 | 1.92 | 1.17-3.14 | 92 | 102 | 1.60 | 0.80-3.20 | 293 | 266 | 2.51 | 1.66-3.79 | 0.12 |
| ≥10 | 63 | 90 | 2.39 | 1.32-4.35 | 31 | 44 | 1.38 | 0.60-3.17 | 109 | 95 | 3.47 | 2.09-5.75 |  |
| Continuous |  |  | 1.02 | 1.00-1.04 |  |  | 1.02 | 0.99-1.04 |  |  | 1.04 | 1.02-1.06 | 0.18 |
| **Age at first exposure, years**^c^ | |  |  |  |  |  |  |  |  |  |  |  |  |
| No exposure | 48 | 86 | 0.61 | 0.36-1.02 | 27 | 37 | 0.67 | 0.33-1.37 | 78 | 106 | 0.48 | 0.31-0.74 |  |
| ≥20 | 115 | 196 | 1 |  | 67 | 89 | 1 |  | 200 | 219 | 1 |  | 0.2 |
| <20 | 137 | 169 | 1.31 | 0.89-1.92 | 77 | 86 | 0.94 | 0.55-1.63 | 257 | 200 | 1.29 | 0.94-1.78 |  |
| Continuous |  |  | 1.00 | 0.98-1.02 |  |  | 1.00 | 0.98-1.03 |  |  | 1.00 | 0.98-1.01 | 0.9 |
| **According to first full-term pregnancy (FFTP)**^c^ | | |  |  |  |  |  |  |  |  |  |  |  |
| Only after FFTP^d^ | 70 | 90 | 1 |  | 39 | 40 | 1 |  | 110 | 111 | 1 |  |  |
| Before (incl. no FTP) | 186 | 288 | 0.76 | 0.49-1.22 | 110 | 139 | 0.66 | 0.35-1.25 | 354 | 319 | 1.00 | 0.68-1.45 |  |

Abbreviations: OR (95%CI): odds ratio (95% confidence interval)

**^a^** Adjusted for age at censoring, birth cohort (≤1945; 1946-1959; ≥1960), number of full-term pregnancies (>2; 1-2; 0), mammography use (never; ever), educational level (intermediate/high; basic; not graduated), BMI (18.5-24.99; <18.5; ≥25 and <30; ≥30), smoking (no; current; past), plus two other DNA repair rare variants groups (when analysis by group of DNA repair rare variant)

^b^ p-value for heterogeneity test.

^c^ Adjusted as in **^a^** plus number of exposures (≤5;>5).

^d^ After = also includes chest X-ray exposure that occurred during the same year of first full-term pregnancy.

**^e^** at least one variant in a gene from the ‘Low’ Group; **^f^** at least one variant in a gene from the ‘No Effect’ Group; **^g^** at least one variant in a gene from the ‘High’ Group.

**Supplemental Table 12.** Sensitivity analyses by variants group excluding variants from the ‘High’ Group from genes individually statistically (or borderline) associated with an increased risk of breast cancer in the GENESIS population (20).

|  | **DNA repair rare variants** | | | | | | | | | | | | | | | |
| --- | --- | --- | --- | --- | --- | --- | --- | --- | --- | --- | --- | --- | --- | --- | --- | --- |
|  | **‘Low’ Group ^e^** | | | | **‘No Effect’ Group ^f^** | | | | **‘High’ Group ^g^** | | | |  | | | |
|  | **Number of** | |  |  | **Number of** | |  |  | **Number of** | |  |  |  | **Number of** | |  |
|  | **Cases** | **Ctrls** | **OR^a^** | **95%CI** | **Cases** | **Ctrls** | **OR^a^** | **95%CI** | **Cases** | **Ctrls** | **OR^a^** | **95%CI** | **Cases** | **Ctrls** | **OR^a^** | **95%CI** |
|  | **OR<0.9** | | | | **0.9≤OR≤1.1** | | | | **OR>1.1^c^** | | | | ***atm+chek2+palb2* ^h^** | | | |
| **Chest X-ray exposure** | | |  |  |  |  |  |  |  |  |  |  |  |  |  |  |
| No | 34 | 67 | 1 |  | 61 | 81 | 1 |  | 57 | 86 | 1 |  | 13 | 12 | 1 |  |
| Yes | 234 | 371 | 1.95 | 1.12-3.40 | 354 | 416 | 1.61 | 1.02-2.52 | 434 | 369 | 3.13 | 1.98-4.97 | 117 | 59 | 3.71 | 1.24-11.1 |
| **Number of exposures** | | |  |  |  |  |  |  |  |  |  |  |  |  |  |  |
| 0 | 34 | 67 | 1 |  | 61 | 81 | 1 |  | 57 | 86 | 1 |  | 13 | 12 | 1 |  |
| <10 | 119 | 183 | 1.96 | 1.10-3.51 | 184 | 215 | 1.52 | 0.94-2.44 | 230 | 209 | 2.85 | 1.76-4.61 | 61 | 29 | 3.02 | 0.96-9.52 |
| ≥10 | 47 | 75 | 2.14 | 1.06-4.31 | 67 | 85 | 2.03 | 1.14-3.64 | 81 | 67 | 3.83 | 2.12-6.93 | 19 | 14 | 3.60 | 0.87-15.0 |
| Continuous |  |  | 1.01 | 0.98-1.04 |  |  | 1.02 | 1.00-1.04 |  |  | 1.04 | 1.01-1.06 |  |  | 1.05 | 0.98-1.11 |
|  | **OR<0.9** | | | | **0.9≤OR≤1.1** | | | | **OR>1.1^d^** | | | | ***atm+chek2+palb2***  ***+fancm+mast1* ^i^** | | | |
| **Chest X-ray exposure** | | |  |  |  |  |  |  |  |  |  |  |  |  |  |  |
| No | 34 | 67 | 1 |  | 61 | 81 | 1 |  | 55 | 78 | 1 |  | 15 | 21 | 1 |  |
| Yes | 234 | 371 | 1.95 | 1.12-3.39 | 354 | 416 | 1.60 | 1.02-2.52 | 415 | 356 | 2.80 | 1.75-4.48 | 152 | 77 | 6.60 | 2.55-17.1 |
| **Number of exposures** | | |  |  |  |  |  |  |  |  |  |  |  |  |  |  |
| 0 | 34 | 67 | 1 |  | 61 | 81 | 1 |  | 55 | 78 | 1 |  | 15 | 21 | 1 |  |
| <10 | 119 | 183 | 1.95 | 1.09-3.50 | 184 | 215 | 1.51 | 0.94-2.44 | 224 | 203 | 2.58 | 1.58-4.21 | 76 | 38 | 5.39 | 1.97-14.8 |
| ≥10 | 47 | 75 | 2.11 | 1.04-4.25 | 67 | 85 | 2.00 | 1.12-3.58 | 75 | 61 | 3.61 | 1.97-6.63 | 27 | 20 | 6.29 | 1.91-20.7 |
| Continuous |  |  | 1.01 | 0.98-1.04 |  |  | 1.02 | 1.00-1.04 |  |  | 1.04 | 1.01-1.06 |  |  | 1.05 | 0.99-1.10 |
|  |  |  |  |  |  |  |  |  |  |  |  |  |  |  |  |  |

Abbreviations: OR (95%CI): odds ratio (95% confidence interval)

**^a^** Adjusted for age at censoring, birth cohort (≤1945; 1946-1959; ≥1960), number of full-term pregnancy (>2; 1-2; 0), mammography use (never; ever), educational level (intermediate/high; basic; not graduated), BMI (18.5-24.99; <18.5; ≥25 and <30; ≥30), smoking (no; current; past), three other DNA repair rare variants groups.

^b^ p-value for heterogeneity test.

^c^ excluded:  *ATM, CHEK2* and *PALB2.*

^d^ excluded: *ATM, CHEK2, PALB2, FANCM* and *MAST1.*

**^e^** at least one variant in a gene from the ‘Low’Group; **^f^** at least one variant in a gene from the ‘No Effect’ Group; **^g^** at least one variant in a gene from the ‘High’Group; **^h^** at least one variant in *atm, chek2* and *palb2;* **^i^** at least one variant in *atm, chek2, palb2,* *FANCM* and *MAST1*.

.
